# Supplementary material for: Strategies to improve the design of gapmer antisense oligonucleotide on allele-specific silencing
Source: Mol Ther Nucleic Acids. 2024 Jun 5;35(3):102237. doi: 10.1016/j.omtn.2024.102237 (PMC11238192; doi:10.1016/j.omtn.2024.102237)
Supplement: Document S1. Figures S1–S6 and Table S1 [file mmc1.pdf]

OMTN, Volume *35*

Supplemental information

Strategies to improve the design of gapmer antisense oligonucleotide on allele-specific silencing

Sara Aguti, Shuzhi Cheng, Pierpaolo Ala, Sean Briggs, Francesco Muntoni, and Haiyan Zhou

Table S1: R^2 values.

R^2 value for dose-response study using 2’-OMe, 2’-MOE, LNA oligos, mixmer and mismatch-mixmer oligos.

	**2'-OMe-1**	**2'-OMe-2**	**2'-OMe-3**	
**WT COL6A3**	0.8541	0.9035	0.7723	
**Mut COL6A3**	0.9772	0.9439	0.9853	
	**2'-MOE-2**	**2'-MOE-3**		
**WT COL6A3**	0.8478	0.9714		
**Mut COL6A3**	0.9696	0.9968		
	**LNA-1**	**LNA-2**		
**WT COL6A3**	0.7882	0.8078		
**Mut COL6A3**	0.8066	0.9382		
	**M-1**	**M-2**	**M-3**	**M-4**
**WT COL6A3**	0.3765	0.5339	-0.4405	-2.86
**Mut COL6A3**	0.9533	0.9367	0.6478	0.6632
	**M-5**	**M-6**	**M-7**	
**WT COL6A3**	-0.2134	0.07064	0.8294	
**Mut COL6A3**	0.7049	0.7014	0.9719	
	**MM (1-7)**	**MM (2-7)**	**MM (3-7)**	**MM (4-7)**
**WT COL6A3**	0.5299	0.5797	0.4955	0.5687
**Mut COL6A3**	0.9439	0.9843	0.934	0.9807
	**MM (5-7)**	**MM (6-7)**		
**WT COL6A3**	0.5946	0.4145		
**Mut COL6A3**	0.9038	0.9616		

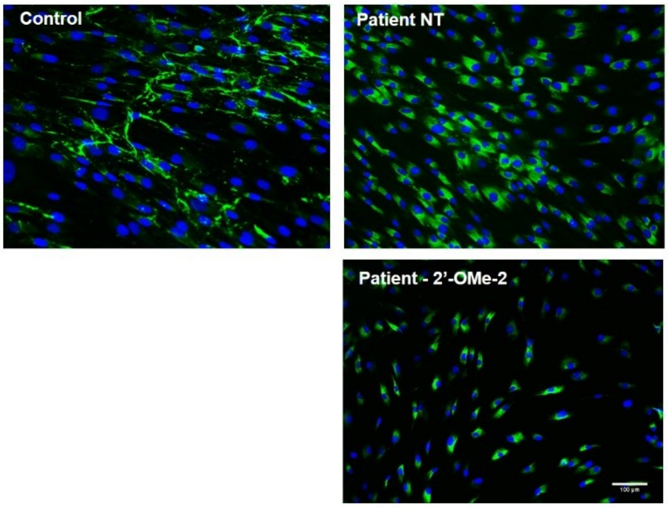

Figure S1: Efficiency of 2’-OMe-2 in restoring ECM collagen VI protein.

Representative images of immunofluorescence staining of collagen VI protein (in green) and nuclei (in blue) in control and patient skin fibroblasts treated with 2’-OMe-2. Pictures were captured under fluorescence microscopy at 10x magnification. Scale bars: 100 µm.

COL6A3 WT

Seq. length		2500 bp	2000bp	1500bp	1000bp	500bp	200bp
N. structures		28	31	37	42	12	5
Exon 15	C	0	1	3	7	0	0
	G	0	1	3	7	0	5
	G	24	28	32	33	12	0
	G	0	0	2	6	2	0
	C	0	0	1	2	0	0
	C	3	2	1	0	0	0
	A	0	1	1	1	0	5
	A	24	29	34	40	11	5
	A	24	30	36	42	12	5
	G	24	30	35	37	12	0
Exon 16	G	0	2	4	8	1	0
	G	0	0	0	0	0	0
	U	0	0	1	0	0	0
	A	0	1	2	1	0	0
	U	3	3	5	9	1	5
	U	27	27	30	29	11	0
	C	1	0	0	1	0	0
	C	0	0	0	1	0	0
	U	0	2	1	2	1	0
	G	1	0	0	1	0	0
	G	0	1	5	10	0	0
	A	1	0	3	6	0	0
	G	0	2	4	8	1	0
	A	3	2	3	4	0	3
	A	28	30	35	31	10	5
	G	28	31	35	37	11	0
	A	3	0	1	3	2	0

COL6A3 Δ16

Seq. length		2500 bp	2000bp	1500bp	1000bp	500bp	200bp
N. structures		30	30	33	35	19	5
Exon 15	C	1	1	4	5	7	0
	G	12	10	14	18	11	3
	G	0	1	3	5	6	0
	G	0	0	0	0	0	0
	C	7	5	3	1	0	0
	C	0	2	2	7	3	0
	A	12	12	15	25	16	3
	A	20	15	29	35	19	5
	A	20	15	29	34	18	5
	G	8	5	14	17	7	2
Exon 17	G	8	3	13	11	5	2
	G	8	3	13	10	3	2
	U	8	3	13	10	3	2
	G	27	21	32	30	17	5
	A	18	21	23	24	14	2
	G	15	18	20	22	13	2
	C	8	3	13	11	4	2
	G	20	13	27	23	10	5
	U	3	10	3	0	3	0
	G	0	0	2	0	0	0
	G	0	0	1	1	0	0
	U	15	11	10	12	6	3
	C	19	13	12	11	5	3
	C	15	12	14	16	10	3
	G	8	5	9	11	2	0
	C	0	0	0	1	1	0
	C	4	2	2	1	1	0

Figure S2: Heat map on the predicted secondary structures of the targeting mRNA sequences

The number of UNAfold-predicted secondary structures is showed for each sequence length, along with ss-count for wild-type mRNA (left lane) and mutant mRNA (right lane).

Nucleotides were color-coded based on the probability of closed structures: >80% in red, 60-80% in light brown, 40-60% in yellow, 20-40% in light green, and <20% in green.

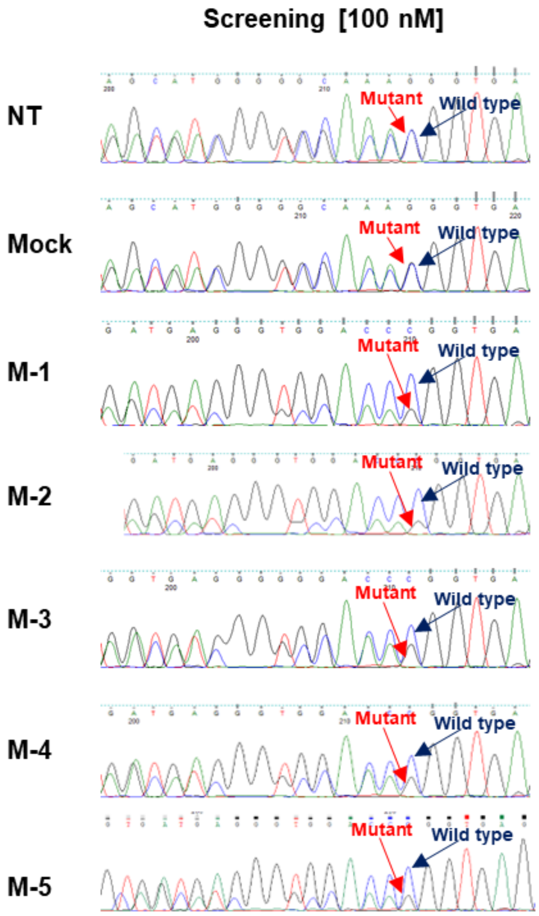

Figure S3: The efficiency of Mixmer ASOs in selectively silencing the mutant allele

Chromatogram of Sanger sequencing of PCR products amplified from UCMD fibroblasts untreated or treated with Mixmer oligos at 100 nM for 24 h. The peaks of wild-type and mutant alleles are indicated with the blue and red arrows.

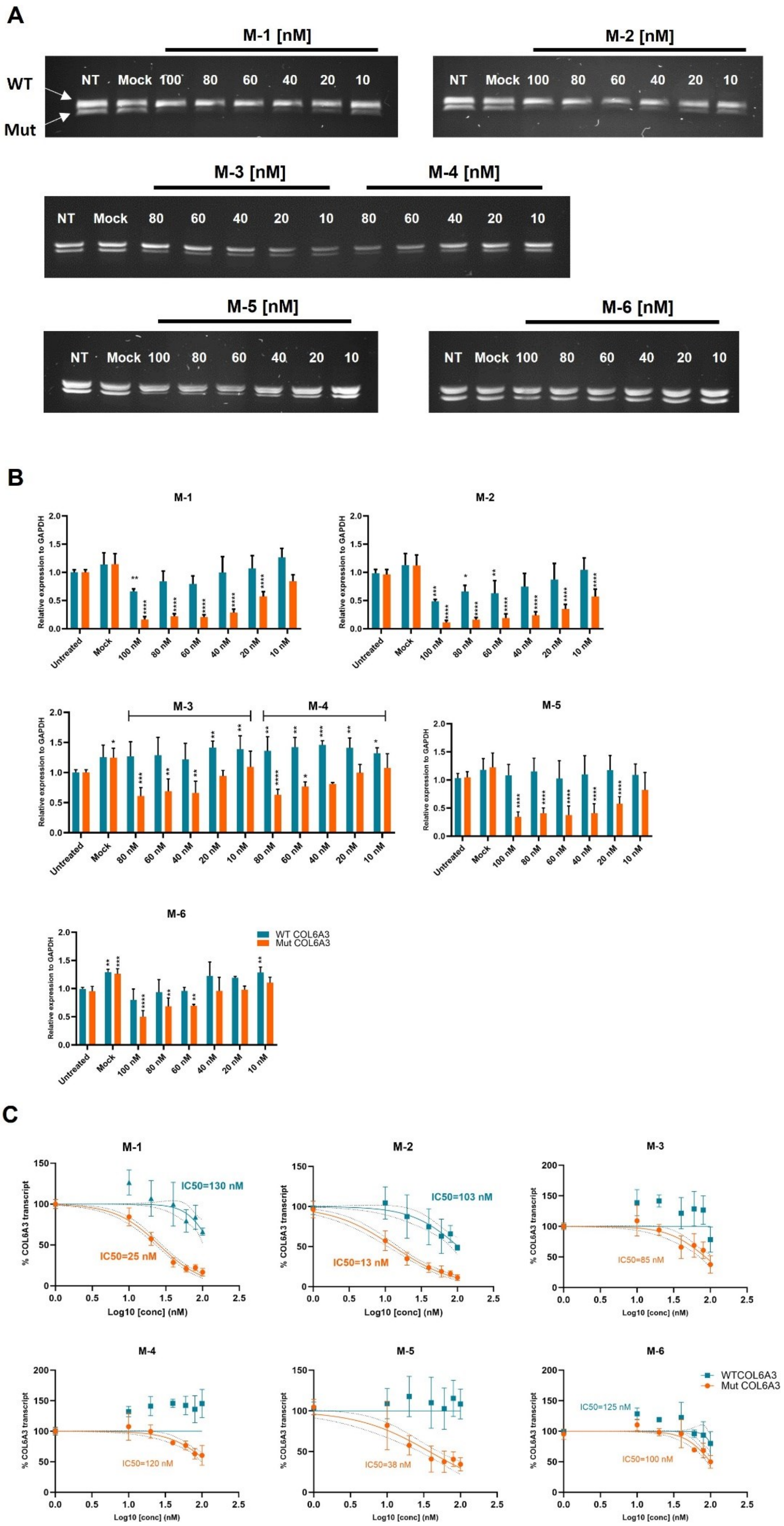
A
M-1 [nM]
M-2 [nM]
WT
Mut
NT
Mock
100
80
60
40
20
10
M-3 [nM]
M-4 [nM]
M-5 [nM]
M-6 [nM]
B
M-1
M-2
M-3
M-4
M-5
M-6
Relative expression to GAPDH
Untreated
Mock
100 nM
80 nM
60 nM
40 nM
20 nM
10 nM
WT COL6A3
Mut COL6A3
C
IC50=130 nM
IC50=25 nM
IC50=103 nM
IC50=13 nM
IC50=85 nM
IC50=120 nM
IC50=38 nM
IC50=125 nM
IC50=100 nM
% COL6A3 transcript
Log10 [conc] (nM)
WTCOL6A3
Mut COL6A3

Figure S4: Dose-response studies of mixmer ASOs on the expression of mutCOL6A3 and wtCOL6A3

UCMD fibroblasts were treated with mixmer ASOs at 100 nM for 24 h, and measured at mRNA level by (A) RT-PCR, (B) allele-specific qRT-PCR on WT and Mut COL6A3 transcripts, respectively. NT= untreated controls, and Mock= cells treated with Lipofectamine 2000 only. Data were normalized to untreated controls and analyzed by one-way ANOVA and post-Bonferroni test. Data are presented as mean ± SD (*$p \leq 0.05$; **$p \leq 0.01$; ***$p \leq 0.001$). (C) Dose-response curve showing IC50 based on the quantification of WT and mutant COL6A3 transcripts.

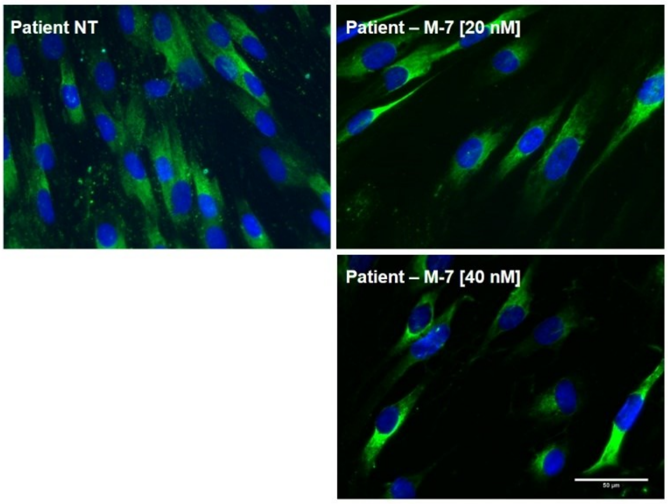

Figure S5: Efficiency of M-7 in restoring ECM collagen VI protein.

Representative images of immunofluorescence staining of COL6 protein (in green) and nuclei (in blue) in untreated patient skin fibroblasts and patient skin fibroblasts treated with M-7 at 20 nM and 40 nM concentration. Fibroblasts were permeabilized using 0.05% Triton X-100. Pictures were captured under fluorescence microscopy at 40x magnification. Scale bars: 50 µm.

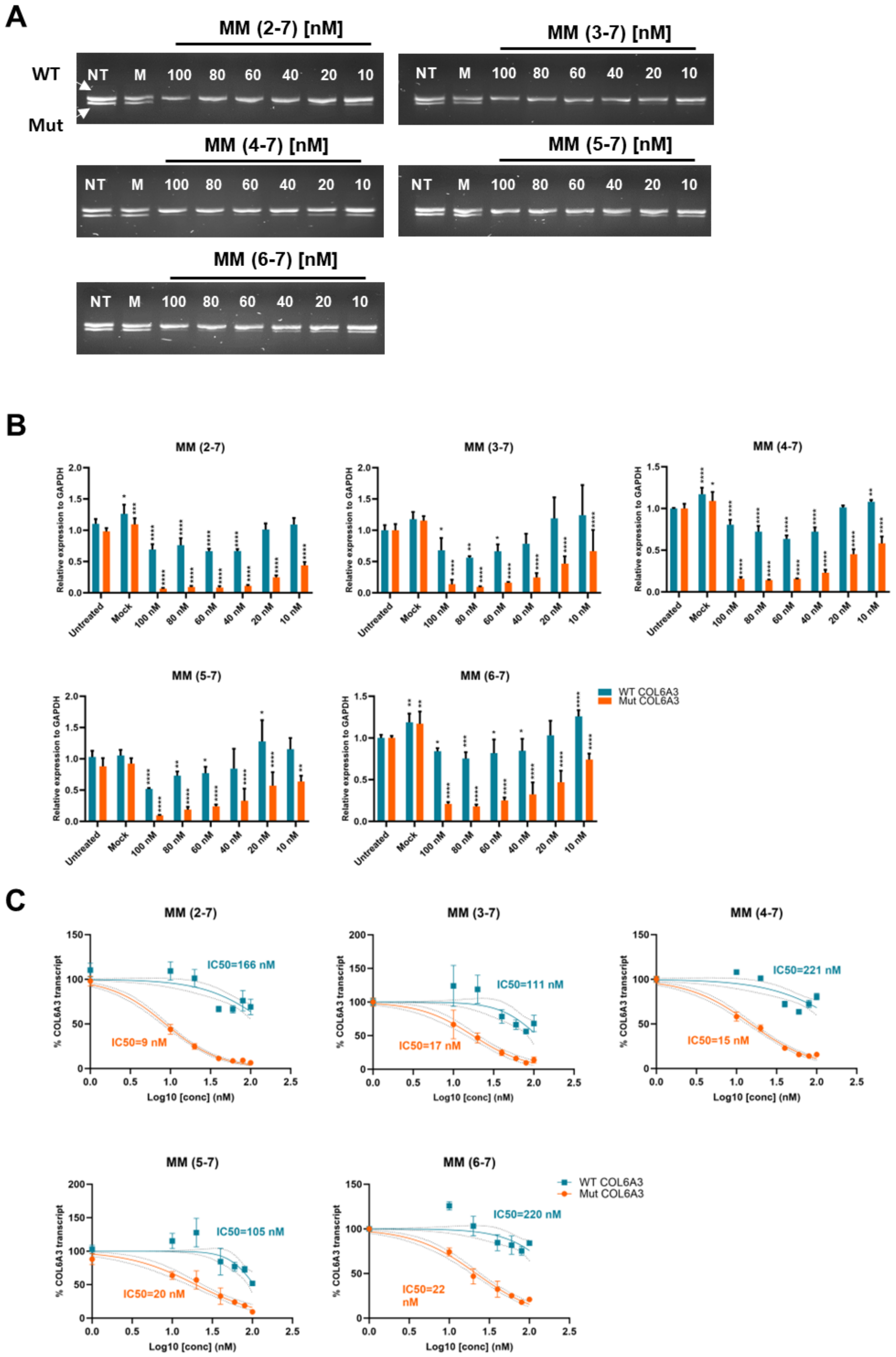

Figure S6: Dose-response studies of mismatch-mixmer ASOs on the expression of mutCOL6A3 and wtCOL6A3.

UCMD fibroblasts were treated with mismatch-mixmer ASOs at 100 nM for 24 h, and measured at mRNA level by (A) RT-PCR, (B) allele-specific qRT-PCR on WT and Mut

COL6A3 transcripts, respectively. NT= untreated controls, and Mock= cells treated with Lipofectamine 2000 only. Data were normalized to untreated controls and analyzed by one-way ANOVA and post-Bonferroni test. Data are presented as mean ± SD ($*p \leq 0.05$; $**p \leq 0.01$; $***p \leq 0.001$). (C) Dose-response curve of showing IC50 based on the quantification of WT and mutant COL6A3 transcripts.
