# Supplementary material for: Strategies to improve the design of gapmer antisense oligonucleotide on allele-specific silencing
Source: Mol Ther Nucleic Acids. 2024 Jun 5;35(3):102237. doi: 10.1016/j.omtn.2024.102237 (PMC11238192; doi:10.1016/j.omtn.2024.102237)
Supplement: Document S2. Article plus supplemental information [file mmc2.pdf]

Molecular Therapy
Nucleic Acids
Original Article

Strategies to improve the design of gapmer antisense oligonucleotide on allele-specific silencing

Sara Aguti,[1] Shuzhi Cheng,[2] Pierpaolo Ala,[3] Sean Briggs,[2] Francesco Muntoni,[1,3,4] and Haiyan Zhou[2,4]

[1]Neurodegenerative Diseases Department, UCL Queen Square Institute of Neurology, London WC1N 3BG, UK; [2]Genetics and Genomic Medicine Research and Teaching Department, Great Ormond Street Institute of Child Health, University College London, London WC1N 1EH, UK; [3]Developmental Neurosciences Research and Teaching Department, Great Ormond Street Institute of Child Health, University College London, London WC1N 1EH, UK; [4]NIHR Great Ormond Street Hospital Biomedical Research Centre, London WC1N 1EH, UK

Gapmer antisense oligonucleotides (ASOs) hold therapeutic promise for allele-specific silencing, but face challenges in distinguishing between mutant and wild-type transcripts. This study explores new design strategies to enhance ASO specificity, focusing on a common dominant mutation in *COL6A3* gene associated with Ullrich congenital muscular dystrophy. Initial gapmer ASO design exhibited high efficiency but poor specificity for the mutant allele. We then adopted a mixmer design, incorporating additional RNA bases based on computational predictions of secondary structures for both mutant and wild-type alleles, aiming to enhance ASO accessibility to mutant transcripts. The mixmer ASO design demonstrated up to a 3-fold increase in specificity compared with the classical gapmer design. Further refinement involved introducing a nucleotide mismatch as a structural modification, resulting in a 10-fold enhancement in specificity compared with the gapmer design and a 3-fold over the mixmer design. Additionally, we identified for the first time a potential role of the RNA-induced silencing complex (RISC), alongside RNase H1, in gapmer-mediated silencing, in contrast with what was observed with mixmer ASOs, where only RNase H1 was involved. In conclusion, this study presents a novel design concept for allele-specific ASOs leveraging mRNA secondary structures and nucleotide mismatching and suggests a potential involvement of RISC in gapmer-mediated silencing.

INTRODUCTION

The clinical development of antisense oligonucleotides (ASOs) to modulate gene transcription began 30 years ago and since then, ten ASO drugs have been approved by the U.S. Food and Drug Administration.[1,2] Among them, five are gapmer ASOs (Fomivirsen,[3,4] Mipomersen,[5,6] Inotersen,[7] Valonesorsen,[8,9] and Tofersen[10]). However, these gapmer ASOs do not discriminate between the mutant and the wild-type alleles. While in certain cases a general down-regulation of a target protein may be desirable, for specific genes this could be detrimental, and a selective lowering of the mutation-containing protein is preferable. To address this, allele-specific silencing approaches using gapmer ASOs have been explored. Discrimination between the wild-type and the mutant alleles can be achieved through a single nucleotide polymorphism (SNP) in *cis* with the mutation, or directly targeting the heterozygous mutations.[11–21] However, due to the sequence similarity and the complexity of secondary structures of the wild-type and mutant transcripts, designing a gapmer ASO that specifically targets the mutant transcripts without affecting the expression of wild-type transcripts can be challenging.[22]

In this study, we have tested two design strategies to enhance the specificity of gapmer ASOs for mutant allele-specific targeting. We evaluated the efficiency of this novel ASO design in correcting a common dominant mutation in *COL6A3* gene (c.6210 + 1G>A) associated with Ullrich congenital muscular dystrophy (UCMD). UCMD represents the severe form of collagen VI-related congenital muscular dystrophy (COL6-CMD), a rare neuromuscular condition caused by dominant or recessive mutations in one of the genes coding for the collagen VI protein (*COL6A1*, *COL6A2*, and *COL6A3*).[23–27] In the last few years, several proof-of-concept studies have been conducted on different ASO strategies for the dominant variants.[28–30] Given that haploinsufficiency is not pathogenic in COL6-CMD, we and others have explored the use of an allele-specific silencing approach, by either small interfering RNA (siRNA) or gapmer ASOs to correct common dominant mutations in COL6-CMD.[31–34] c.6210 + 1G>A mutation in *COL6A3* gene leads to an *in-frame* exon 16 deletion in the mature transcripts. We designed and tested various classical gapmer ASOs with different chemical modifications, including 2′-O-methyl (2′-OMe), 2′-O-methoxyethyl (2′-MOE), and locked nucleic acid (LNA), in patient-derived dermal fibroblasts. However, despite a high efficiency in silencing, they showed poor specificity in selectively

Received 11 March 2024; accepted 31 May 2024;
https://doi.org/10.1016/j.omtn.2024.102237.

Correspondence: Francesco Muntoni, Neurodegenerative Disease Department, UCL Queen Square Institute of Neurology, London WC1N 3BG, UK.
E-mail: f.muntoni@ucl.ac.uk
Correspondence: Haiyan Zhou, Genetics and Genomic Medicine Research and Teaching Department, Great Ormond Street Institute of Child Health, University College London, London WC1N 1EH, UK.
E-mail: haiyan.zhou@ucl.ac.uk

Published by Elsevier Inc. on behalf of The American Society of Gene and Cell Therapy.
This is an open access article under the CC BY license (http://creativecommons.org/licenses/by/4.0/).

targeting the mutant transcripts. This low specificity was likely due to the high sequence similarity between the wild-type and mutant alleles. Therefore, to enhance specificity while maintaining high efficiency, we modified the gapmer ASO design in two steps. Gapmer ASO is designed as chimeric oligonucleotides containing a central block of phosphorothioate (PS)-modified DNA (DNA gap) flanked by chemically modified ribonucleotides (RNA wings). Based on the understanding that in gapmer ASOs the binding affinity with the target RNA is primarily attributed to the RNA wings,[35,36] we introduced additional RNA nucleotides into the DNA gap to potentially enhance target binding affinity,[37–39] following the mixmer design, which consists of an alternation of RNA and DNA nucleotides.[39–41] These additional RNA nucleotides were strategically placed based on the secondary structures of the target mRNA, allowing a more favorable ASO accessibility to the mutant allele than to the wild-type allele.[42,43] We specifically selected and placed RNA nucleotides in sequence regions presenting opened or partially opened secondary structures of the mutant allele but closed structures of the wild-type allele. This design did not affect ASO's efficiency in silencing the mutant allele and led to a 3-fold improvement in specificity. The specificity was further enhanced by introducing a single nucleotide mismatch complementary to a nucleotide in exon 15 found in the open secondary structure in both wild-type and mutant sequences, to tune down the binding affinity to the wild-type allele.

Following these modifications, a 10-fold improvement compared with the classical gapmer design, and 3-fold compared with the mixmer design, was observed. In conclusion, our findings provide a novel design concept for allele-specific ASOs with improved silencing specificity based on the target mRNA's secondary structures and the introduction of nucleotide mismatching.

In addition, our mechanistic investigation revealed further insights into the silencing effects of gapmer and mixmer oligos. For the first time we showed that the silencing effect of gapmer oligos is not solely dependent on RNase H1 activation; instead, it appears that the RNA-induced silencing complex (RISC) is partially involved. In contrast, the silencing effect of mixmer oligos is exclusively dependent on the activation of the RNase H1 enzyme.

RESULTS

Design and systematic screening of classical gapmer ASOs to specifically silence the mutant COL6A3 mRNA

To investigate the selective silencing of gapmer ASOs on the mutant COL6A3 (mutCOL6A3) mRNA, we designed and systematically screened classical gapmer ASOs. The presence of the heterozygous c.6210+1G>A mutation in *COL6A3* gene results in the in-frame skipping of exon 16 from the mature mRNA, creating a novel junction between exons 15 and exon 17 (Figure 1A). This novel junction presents a promising target for gapmer ASOs designed to specifically silence the mutCOL6A3, while leaving wild-type COL6A3 (wtCOL6A3) intact.

We designed a set of 15 gapmer ASOs with lengths ranging from 13- to 23-mer, incorporating a central PS-DNA gap of 7- to 11-mer. These classical gapmer ASOs were designed to be complementary to the sequence of mutCOL6A3, with the PS-DNA gap positioned at the exon 15/17 junction. Surrounding the PS-DNA gap, we included short RNA nucleotides ranging from 3- to 7-mers in length, modified with 2′-OMe, 2′-MOE, or LNA chemical modifications (Table 1).

We first evaluated the efficiency of the classical gapmer ASOs in 2′-OMe chemistry. Skin-derived fibroblasts cultured from two UCMD patients carrying the c.6210+1G>A mutation were treated with ASOs at 100 nM. RT-PCR and RT-qPCR showed a significant reduction in mutCOL6A3 expression with 2′-OMe-1 to -7, except for 2′-OMe-8 and 2′-OMe-9. However, most of the ASOs also significantly suppressed the expression of wtCOL6A3 (Figures 1B and 1C). The ratio of mutCOL6A3 to wtCOL6A3 revealed that the most effective ASOs able to suppress mutCOL6A3 by more than 75% compared with wtCOL6A3 expression was achieved with 2′-OMe-1, -2, and -3 (Figure 1D).

Therefore, to enhance the specificity of the most effective gapmer ASOs to mutCOL6A3 and minimize the suppression effect on the wtCOL6A3, we explored different chemical modifications of the RNA wings. 2′-MOE and LNA chemistries were tested considering their higher binding affinity compared with 2′-OMe chemistry.[1] Fibroblasts were treated with 2′-MOE and LNA ASOs at 100 nM and, as shown in Figure 1C, they exhibited improved efficiency in silencing mutCOL6A3. All ASOs were capable of suppressing mutCOL6A3 expression by more than 75% compared with wtCOL6A3 expression (Figure 1D). However, no improvement in specificity was detected; all ASOs caused a more than 70% decrease in the expression of wtCOL6A3 (Figure 1C).

Classical gapmer oligos suppressed mutCOL6A3 in a dose-dependent manner

The most effective gapmer ASOs, which achieved a more than 75% suppression of the mutCOL6A3 over wtCOL6A3 (2′-OMe-1, 2′-OMe-2, 2′-OMe-3, 2′-MOE-2, 2′-MOE-3, LNA-1, and LNA-2) were additionally studied at lower concentrations. Concentrations ranging between 10 and 80 nM were tested for 2′-OMe oligos and between 1.25 and 20 nM were used for 2′-MOE and LNA oligos. The corresponding R^2 value was presented in Table S1. The reason for further decreasing the ASOs concentration was to minimize their off-target silencing effects on wtCOL6A3. However, lower concentrations of gapmer ASOs did not alleviate the off-target suppression on wtCOL6A3, as similar median inhibition concentration (IC_{50}) values were detected between the mutant and wtCOL6A3 (Figure 2).

Among all the tested ASOs, 2′-OMe-2 demonstrated a better ability in differentiating between wild-type and mutCOL6 transcripts. Therefore, its efficiency in restoring COL6 at protein level was further tested in patients skin fibroblasts carrying the c.6210+1G>A mutation. No improvements in collagen VI protein structure and localization were observed. Untreated patient fibroblasts displayed cytoplasm accumulation of collagen VI protein. However, ASO treatment in patient fibroblasts did not yield any restoration of collagen VI protein

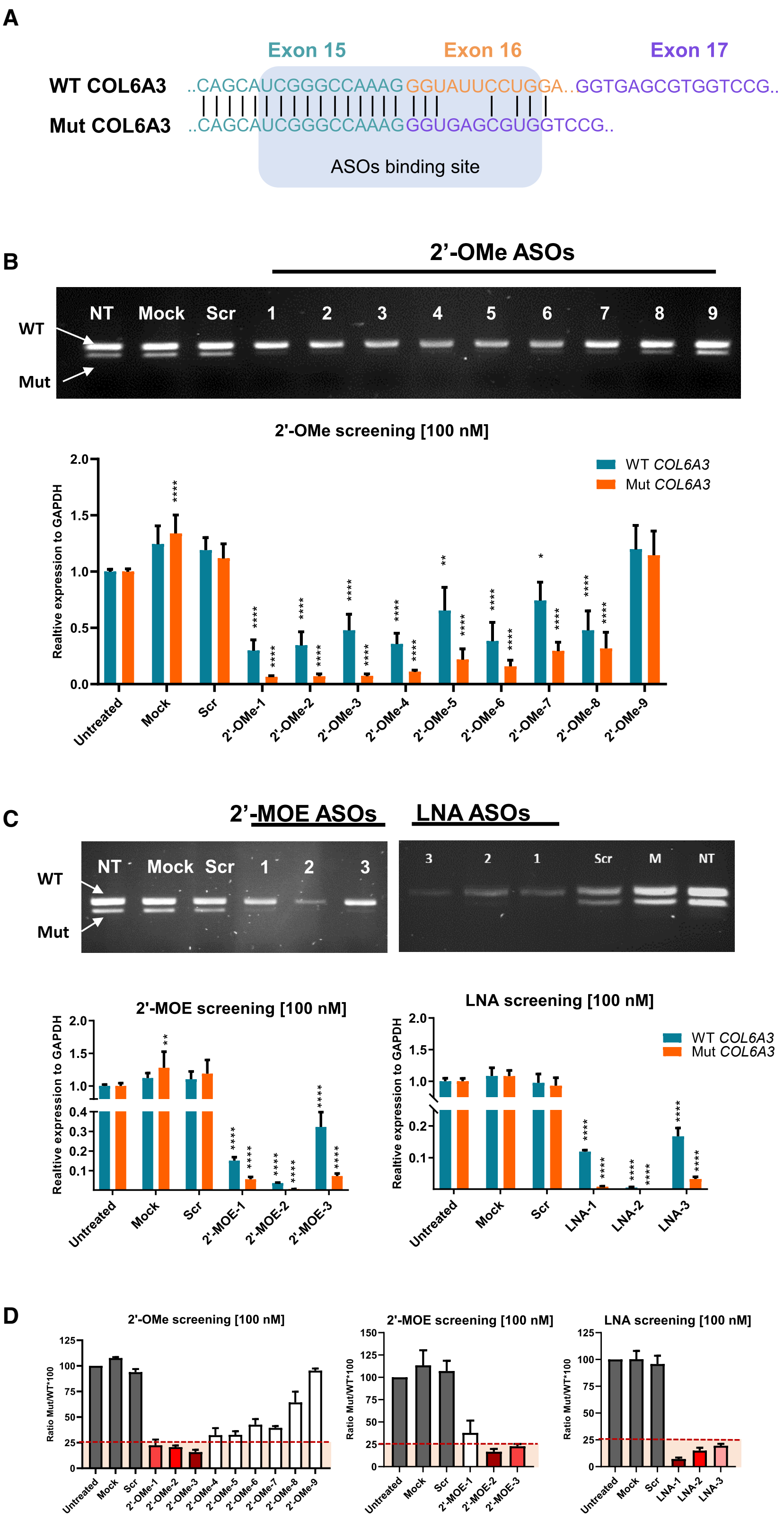

Figure 1. Evaluation of 2′-OMe, 2′-MOE, and LNA gapmer ASOs on the expression of mutant and wild-type COL6A3 mRNAs

(A) Schematic representation of wild-type (WT) and mutant (Mut) COL6A3 sequences and the ASOs' binding site. (B) Gel electrophoresis and allele-specific RT-qPCR from samples isolated from UCMD fibroblasts treated with 2′-OMe ASOs at 100 nM for 24 h with Lipofectamine transfection. Mock, cells treated with Lipofectamine 2000 only; NT, untreated control; Scr, cells treated with scrambled ASO. RT-qPCR data were normalized to untreated samples and analyzed by one-way ANOVA and post-Bonferroni test. Data are presented as mean ± SD (*$p \leq 0.05$; **$p \leq 0.01$; ***$p \leq 0.001$). (C) Gel electrophoresis and allele-specific RT-qPCR from samples isolated from UCMD fibroblasts treated with 2′-MOE and LNA ASOs at 100 nM for 24 h with Lipofectamine transfection. RT-qPCR data were normalized to untreated samples and analyzed by one-way ANOVA and post-Bonferroni test. Data are presented as mean ± SD (*$p \leq 0.05$; **$p \leq 0.01$; ***$p \leq 0.001$). (D) Ratio of mutCOL6A3 to wtCOL6A3. ASOs below the red dashed line were those able to suppress mutCOL6A3 by more than 75% compared with wtCOL6A3 expression.

Table 1. List of ASOs designed to target the mutCOL6A3 mRNA

							Binding affinity		
	5'–>3'	Length	DNA gap	GC%	Tm	Off-target	Mutant (kcal/M)	Wild-type (kcal/mol)	
2′-OMe-1	[**CCACGC**] T*C*A*C*C*C*T*T*T*G* [**GCCCGA**]	22	10	68.2	75.2	no	−34.24	−16.62	
2′-OMe-2	[**CCACGCU**] C*A*C*C*C*T*T*T* [**GGCCCGA**]	22	8	68.2	76.5	no	−34.24	−16.62	
2′-OMe-3	[**ACCACG**] C*T*C*A*C*C*C*T*T*T* [**GGCCCG**]	22	10	68.2	76.8	no	−34.29	−14.66	
2′-OMe-4	[**ACCACGC**]T*C*A*C*C*C*T*T* [**UGGCCCG**]	22	8	68.2	77.5	no	−34.29	−14.66	
2′-OMe-5	[**ACCACG**] C*T*C*A*C*C*C*T* [**UUGGCC**]	20	8	65	74.2	no	−29.54	−11.44	
2′-OMe-6	[**CACGCU**] C*A*C*C*C*T*T*T* [**GGCCCG**]	20	8	70	72.7	no	−31.34	−14.73	
2′-OMe-7	[**CCACG**] C*T*C*A*C*C*C*T* [**UUGGC**]	18	8	66.7	69.3	yes	−26.58	−11.59	
2′-OMe-8	[**ACGCU**] C*A*C*C*C*T*T*T* [**GGCCC**]	18	8	66.7	70.8	yes	−27.84	−13.54	**Gapmer**
2′-OMe-9	[**ACG**] C*T*C*A*C*C*C*T* [**UUG**]	14	8	57.1	55.2	yes	−20.07	−11.43	
2′-MOE-1	<**CCACGCT**> C*A*C*C*C*T*T*T* <**GGCCCGA**>	22	8	68.2	64.5	no	−34.24	−16.62	
2′-MOE-2	<**ACCACG**> C*T*C*A*C*C*C*T*T*T* <**GGCCCG**>	23	11	65.2	66	no	−34.89	−14.66	
2′-MOE-3	<**CACG**> C*T*C*A*C*C*C*T*T*T* <**GGCC**>	18	9	66.7	57.8	yes	−26.59	−10.06	
LNA-1	{**CGC**} T*C*A*C*C*C*T* {**TTG**}	13	7	61.5	58.2	yes	−18.45	−11.43	
LNA-2	{**CAC**} G*C* {**T**} C*A*C*C*C*T*T*T* {**G**} G*C* {**CCG**}	19	8	70	75.6	no	−27.48	−11.42	
LNA-3	{**ACG**} C*T*C*A*C*C*C*T*T*T* {**GGC**}	16	10	62.5	63.9	yes	−22.05	−10.56	
M-1	[**CCA**] C*G*C* [**UCA**] C*C*C* [**UUU**] G*G*C* [**CCG**]	21	3	71.4	56.1	no	−33.71	−14.70	
M-2	[**GGC**] G*G*A* [**CCA**] C*G*C* [**UCA**] C*C*C* [**UUU**]	21	3	66.7	58.8	no	−30.37	−21.36	
M-3	[**CA**] C*G*C* [**UCA**] C*C*C* [**UUU**] G*G*C* [**CC**]	19	3	68.4	53.4	yes	−30.15	−13.54	
M-4	[**CCA**] C*G*C* [**UCA**] C*C*C* {**UUU**}	15	3	60	52.2	yes	−22.41	−11.86	**Mixmer**
M-5	[**CCAC**] G*C*T* [**CACCCU**] T*T*G* [**GCCC**]	20	3	70	63.4	yes	−32.52	−13.51	
M-6	[**CUC**] A*C*C* [**CUUU**] G*G*C* [**CCG**]	16	3	68.8	44.3	yes	−23.42	−15.87	
M-7	[**GAC**] C*A*C*G* [**CUC**] A*C*C* [**CUUU**] G*G*C* [**CCG**]	23	4	69.6	60	no	−37.82	−16.17	
MM (1–7)	[**GAC**] C*A*C*G* [**CUC**] A*C*C* [**CUAU**] G*G*C* [**CCG**]	23	4	69.6	63.6	no	−31.49	−15.35	
MM (2–7)	[**GAC**] C*A*C*G* [**CUC**] A*C*C* [**CUUA**] G*G*C* [**CCG**]	23	4	69.6	61.8	no	−31.09	−18.14	
MM (3–7)	[**GAC**] C*A*C*G* [**CUC**] A*C*C* [**CUUU**] C*G*C* [**CCG**]	23	4	69.6	60	no	−29.88	−21.09	**Mismatch Mixmer**
MM (4–7)	[**GAC**] C*A*C*G* [**CUC**] A*C*C* [**CUUU**] G*C*C* [**CCG**]	23	4	69.6	60.5	no	−29.06	−17.72	
MM (5–7)	[**GAC**] C*A*C*G* [**CUC**] A*C*C* [**CUUU**] G*G*G* [**CCG**]	23	4	69.6	60.5	no	−27.46	−12.8	
MM (6–7)	[**GAC**] C*A*C*G* [**CUC**] A*C*C* [**CUUU**] G*G*C* [**GCG**]	23	4	69.6	60.5	no	−28.32	−12.59	

PS-DNA is indicated with *, nucleotides modified with 2′-OMe chemistry are indicated in bold inside square brackets, nucleotides in 2′-MOE chemistry are in bold inside angle brackets, and nucleotides in LNA chemistry are in bold in curly brackets. Mismatch nucleotides are underlined.

within the extracellular matrix (ECM), likely due to the nonspecific silencing effect on both wild-type and mutant transcripts (Figure S1).

The design of mixmer ASOs to enhance the selectivity of ASOs to mutCOL6A3 over wtCOL6A3

The UNAfold software was used to predict the secondary structures of both wtCOL6A3 and mutCOL6A3. To enhance the accuracy of prediction, RNA sequences of both transcripts in various lengths were analyzed, including 200 bp, 500 bp, 1,000 bp, 1,500 bp, 2,000 bp, and 2,500 bp, by placing the junction of exon 15/16 for wtCOL6A3 and the junction of exon 15/17 for mutCOL6A3 in the center of each sequence. Based on the prediction, the software generated the number of secondary structures as a count (ss-count). The ss-count represents the likelihood of a base to be single stranded, measured by the frequency of a nucleotide occurrence in a single strand among the predicted structures. For instance, in the 2,500-bp sequence of mutCOL6A3, UNAfold predicted 30 different structures. Therefore, each nucleotide within this region would have an ss-count ranging from

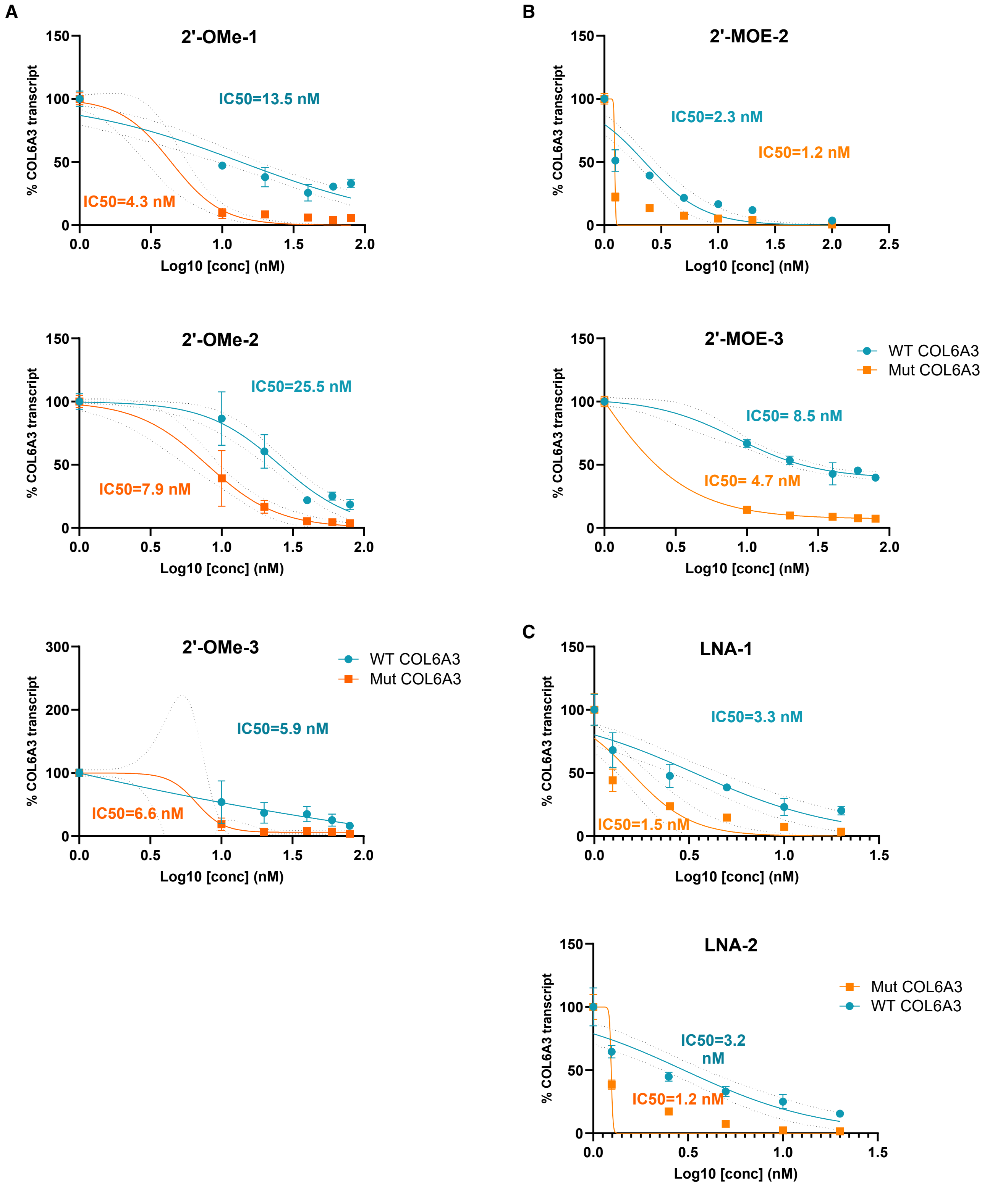

(legend on next page)

0 to 30, where 30 indicates the nucleotide is unbound in all 30 structures, and 0 indicates it is bound to another nucleotide in all predicted structures. Therefore, a higher ss-count represents a more open secondary structure and hence greater accessibility to ASOs.

Based on this information, a heatmap was generated, presenting closed secondary structures (lower ss-counts) in red and open secondary structures (higher ss-counts) in green (Figure S2). Seven mixmer ASOs were designed based on the heatmap by placing the RNA modification in ASO complementary to nucleotides showing an open or semi-open structure in the mutCOL6A3, but a closed structure in the wtCOL6A3, to increase its binding affinity to the mutCOL6A3 over wtCOL6A3 transcripts (Figure 3).

The efficacy and specificity of mixmer ASOs were evaluated at 100 nM using RT-PCR, RT-qPCR, and Sanger sequencing for the readout (Figures 4 and S3). PCR and RT-qPCR results showed a decrease in mutCOL6A3 expression after mixmer ASO treatment, with the greatest suppression observed from M-7 (Figures 4A and 4B). The qPCR results demonstrated a high efficiency of M-1, M-2, and M-7 in silencing mutCOL6A3 expression, resulting in a greater than 75% downregulation when compared with wtCOL6A3 (Figure 4C). However, wtCOL6A3 expression was also affected, although to a lesser extent compared with the classical gapmer ASOs. The average of suppression effects on mutCOL6A3 were similar across gapmer ASOs in 2′-OMe, 2′-MOE, or LNA modification or mixmer, while the suppression on wtCOL6A3 was much lower in mixmer ASOs compared with gapmer ASOs (2′-OMe, 2′-MOE, and LNA). In particular, the downregulation of wtCOL6A3 after mixmer treatment (45%) was 44% less than the LNA gapmer (90%), 36% less than 2′-MOE gapmer (82%), and 17% less than 2′-OMe gapmer (63%) (Figure 4D).

The efficiency of mixmers was further evaluated through dose-response studies by testing ASOs at a range of concentrations from 10 nM to 100 nM (Figures 4E, 4F, and S4). M-1, -2, and -7 exhibited dose-dependent suppression on mutCOL6A3 expression, with IC_{50} at 25 nM, 13 nM, and 7 nM, respectively. The IC_{50} for wtCOL6A3 was 130 nM, 103 nM, and 83 nM, respectively (Figures 4G and S4C). Among these ASOs, M-7 demonstrated the greatest potency, displaying superiority at around 8-fold discrimination between wtCOL6A3 (IC_{50} = 83 nM) and mutCOL6A3 (IC_{50} = 7 nM). The corresponding R^2 values are provided in Table S1.

The efficacy of M-7 treatment was further evaluated at the protein level. Immunofluorescence staining was performed in patient fibroblasts treated with different concentrations of M-7. However, no restoration of collagen VI in the ECM was observed after M-7 treatment (Figure S5). The lack of efficiency on restoring collagen VI protein is likely due to the persistence of off-target effects of M-7 on wtCOL6A3. Although the mixmer design significantly improves ASO's specificity to mutCOL6A3, the residual suppression of wtCOL6A3 still limits its effect at protein level.

Mutant allele discrimination was further improved by introducing nucleotide mismatch in the ASO sequence

To further improve the specificity of mixmer ASOs to mutCOL6A3, a nucleotide mismatch was introduced in the sequence of M-7 as a structural modification. The mismatches were strategically designed to be complementary to exon 15, thereby decreasing the binding affinity between the ASOs and wtCOL6A3. Six new mismatch-mixmer ASOs (MM-ASOs) were designed and tested in patient's fibroblasts (Figure 5A).

As measured by RT-PCR and RT-qPCR, the MM-ASOs exhibited significant efficiency in silencing mutCOL6A3 expression comparable to the original M-7 (Figures 5B and 5C). Interestingly, the MM-ASOs also showed significantly improved specificity with decreased suppression of the wtCOL6A3, ranging from 16% to 48% downregulation, compared with the original M-7, which presented a 52% suppression of wtCOL6A3 (Figure 5D). To determine the optimal concentration for silencing mutCOL6A3 while minimizing the impact on wtCOL6A3, dose-response studies of the 7 MM-ASOs were conducted at a concentration range of 10 nM–100 nM (Figures 5E, 5F, and S6). The corresponding R^2 value is shown in Table S1. All these MM-ASOs demonstrated a dose-dependent suppression on the mutCOL6A3 expression. Among them, MM (1–7) ASO exhibited the highest efficiency and specificity showing an IC_{50} of 8 nM for mutCOL6A3 and 261 nM for wtCOL6A3 (Figure 5G), with a 32-fold discrimination between mutCOL6A3 and wtCOL6A3. The introduction of one mismatch in the MM (1–7) did not affect its efficiency in silencing mutCOL6A3 when compared with the original sequence M-7, as indicated by similar IC_{50} values for mutCOL6A3 (IC_{50} (M–7) = 6.8 nM; IC_{50} (MM (1–7)) = 7.7 nM). However, the specificity of the MM (1–7) was significantly enhanced, exhibiting a 3-fold increase compared with the original M-7 sequence. This was evidenced by the IC_{50} values for wtCOL6A3, with MM (1–7) at 261 nM and M-7 at 83 nM (Figure 5H). Furthermore, when comparing the efficiency and specificity of the MM (1–7) with the best-performing classical gapmer ASOs (2′-OMe-2), no change in ASO silencing efficiency was observed. However, there was a 10-fold increase in specificity in targeting the wtCOL6A3 transcripts (IC_{50} (2′-OMe-2) = 25.5 nM; IC_{50} (MM (1–7)) = 261 nM) (Figure 5H).

The lead MM (1–7) was further evaluated at the protein level by immunostaining of collagen VI in cultured patient fibroblasts. Unlike the original M-7, which did not induce any collagen VI protein secretion in ECM, a single treatment of MM (1–7) at 40 nM clearly presented the deposition of a few linear collagen VI microfibrils in ECM (Figure 6).

Figure 2. Dose-response studies of gapmer ASOs in 2′-OMe, 2′-MOE, and LNA chemistries on the expression of mutCOL6A3 and wtCOL6A3

UCMD patient's fibroblasts were treated with gapmer ASOs at a range of concentrations between 10 nM and 80 nM for 2′-OMe oligos and between 1.25 nM and 20 nM for 2′-MOE and LNA oligos. The IC_{50} was identified on the quantification of wild-type (WT) and mutCOL6A3 transcripts. The dose-response curves of 2′-OMe-1, -2,-3 (A), 2′-MOE-2,-3 (B), and LNA-1,-2 (C) were displayed. Log10 [concentration] was used in calculating IC_{50}.

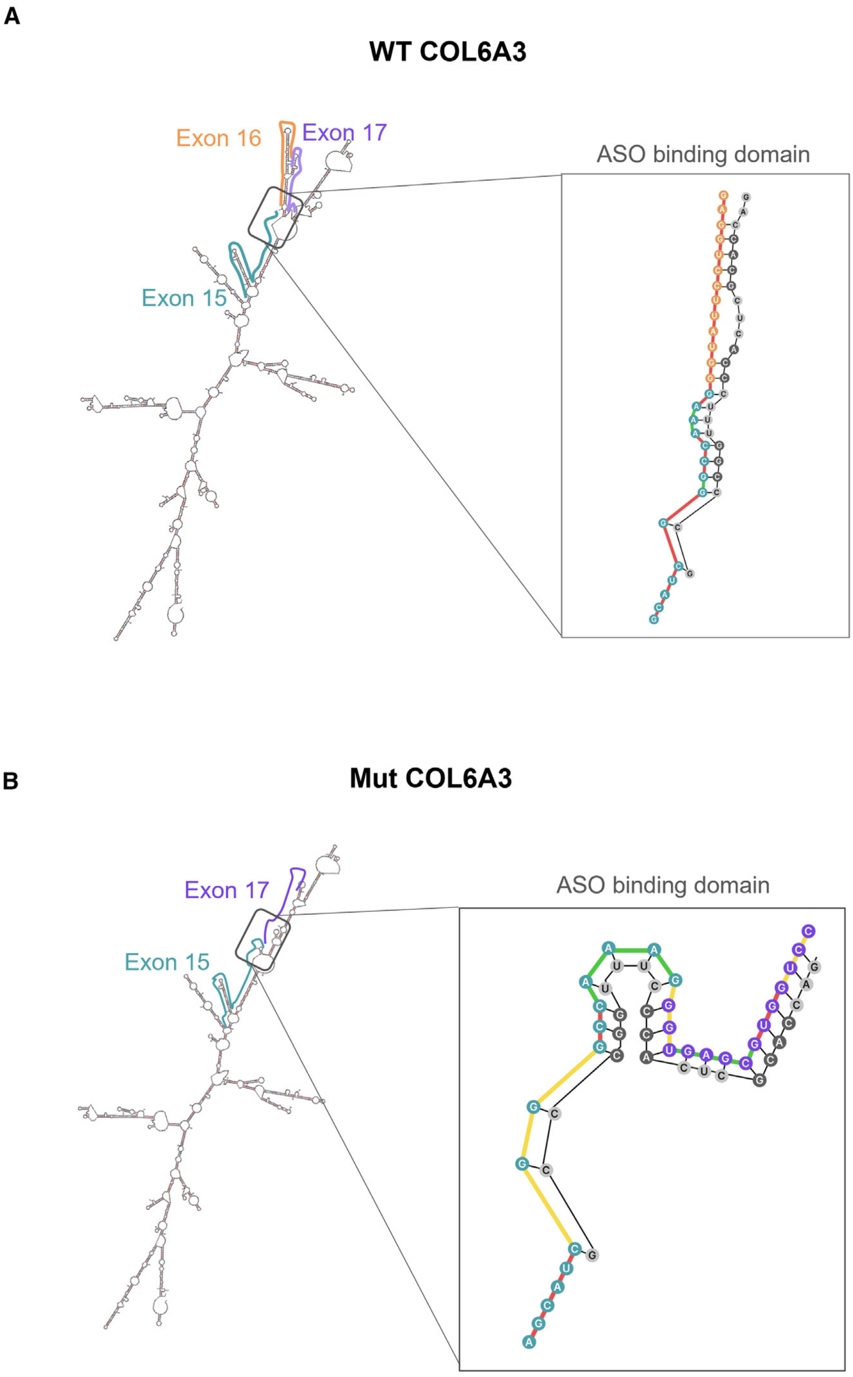

Figure 3. ASO binding domain in WtCOL6A3 and MutCOL6A3 secondary structures

(A) wtCOL6A3 and (B) MutCOL6A3 secondary structures were predicted using UNAFold with 1,500 bp of RNA sequences. In the secondary structures, exon 15 is shown in blue, exon 16 in orange, and exon 17 in purple. A magnification of the ASO binding domain shows the interaction of M-7 with the target RNA. DNA nucleotides are shown in black, while RNA nucleotides are shown in gray. The color of the lines connecting the nucleotides of the wtCOL6A3 and MutCOL6A3 represents open structures (green), semi-open structures (yellow), and closed structures (red).

These findings suggest a potential therapeutic effect of the MM (1–7) ASOs on the restoration of normal collagen VI architecture.

Mixmer ASOs silence COL6A3 via RNase H-mediated RNA cleavage

To investigate the underlying mechanism of action (MOA) responsible for the silencing effect of mixmer ASOs and to confirm whether the observed silencing effect was mediated by RNase H1 activation (typical for gapmer ASOs) or RISC formation (typical for siRNAs), we conducted additional experiments. Aurintricarboxylic acid (ATA) (Figure 7A),[44] a potent inhibitor of RISC assembly, and the RNase H enzyme for cleaving DNA/RNA duplexes were used.

Patient fibroblasts were transfected with either a siRNA to silence COL6A3 expression as a positive control (Figure 7B) or a classical gapmer ASO (LNA-2) or the lead MM-ASO (1–7). ATA inhibitor at 6.25 μM, or DMSO, was added to untreated or treated samples. As expected, ATA treatment fully restored COL6A3 expression in siRNA-treated samples, confirming the RISC-dependent silencing. Interestingly, a slight but significant restoration was also observed in samples treated with gapmer ASOs (Figure 7C), indicating a potential, albeit minimal, involvement of RISC in addition to RNase H, which is the known major MOA of gapmer ASO in gene silencing. However, there was no restoration from ATA inhibitor in mixmer ASO-treated samples, implying that RISC is not involved in gene silencing mediated by mixmer ASOs (Figure 7C).

These findings were further confirmed through an RNase H assay. Initially, two mixmer and two gapmer ASOs were annealed with complementary RNA, followed by 20 min incubation with the RNase H enzyme at room temperature. Clear cleavage was observed upon the addition of the RNase H enzyme to both RNA/gapmer or RNA/mixmer duplexes. However, the cleaved bands in RNA/mixmer duplexes were much stronger than that in RNA/gapmer duplexes (65% vs 14%, Figure 7D), indicating a more active involvement of RNase H in mixmer-induced silencing than in gapmer-induced silencing.

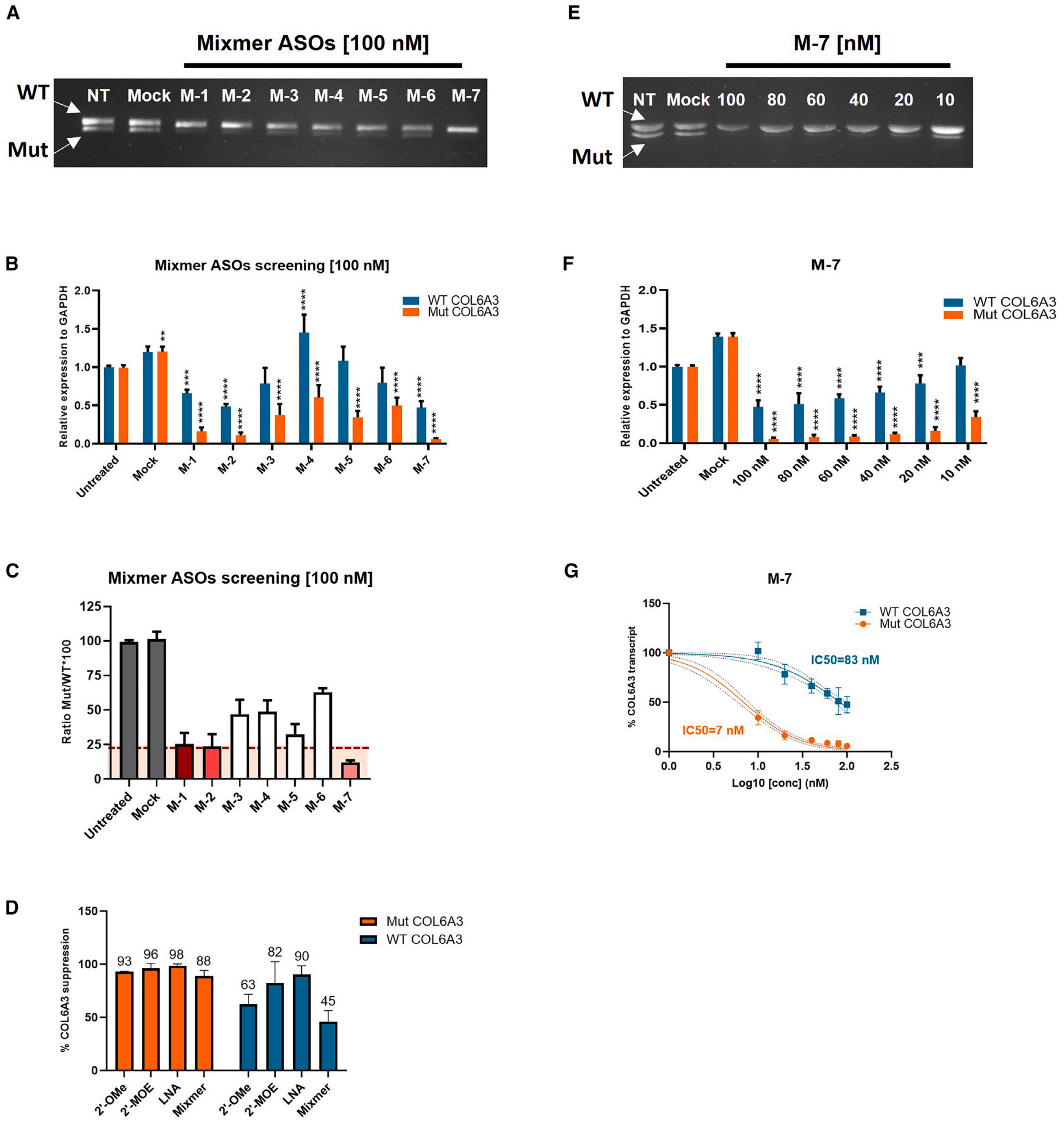

Figure 4. Evaluation of mixmer ASOs on the expression of mutant and wtCOL6A3 mRNAs

UCMD fibroblasts were treated with mixmer ASOs at 100 nM for 24 h with Lipofectamine transfection, and measured at mRNA level by (A) RT-PCR and (B) allele-specific qRT-PCR on wild-type (WT) and Mut COL6A3 transcripts. Mock, cells treated with Lipofectamine 2000 only; NT, untreated controls. Data were normalized to untreated controls and analyzed by one-way ANOVA and post-Bonferroni test. Data are presented as mean ± SD (*$p \leq 0.05$; **$p \leq 0.01$; ***$p \leq 0.001$). (C) Ratio of mutCOL6A3 to wtCOL6A3. Oligos below the red dashed line were those able to suppress mutCOL6A3 by over 75% compared with wtCOL6A3 expression. (D) Average of the mutant (in orange) and wild-type (in blue) suppression after treatment with classical 2′-OMe, 2-MOE, LNA gapmer and mixmer oligos. UCMD fibroblasts were treated with mixmer M-7 ASO at a range of

(legend continued on next page)

DISCUSSION

Allele-specific gene silencing by gapmer ASO is a powerful therapeutic strategy for conditions caused by dominant gain-of-function mutations. The specific discrimination between mutant and wild-type alleles is pivotal in ASO design and has become a challenge hindering the wide application of this technology. Here, we compared three different ASO design strategies for allele-specific silencing, including the classical gapmer, mixmer, and mismatch mixmer. The classical gapmer ASOs efficiently silenced the mutant allele but lacked specificity (Figures 1 and 2). We have explored various RNA chemical modifications in gapmer ASOs, including 2′-OMe, 2′-MOE, and LNA. Among the three chemical modifications, 2′-OMe exhibits the optimal effects in both efficiency and specificity in allele-specific silencing, compared with 2′-MOE and LNA.

Our mixmer ASO design is based on an extensive computational analysis of secondary structure predictions on both mutant and wild-type sequences, to identify short open sequence regions, more accessible to ASO binding in the mutant transcripts than in the wild-type transcripts. When we designed ASO complementary to the target mRNA, we chose to put the ASO sequence targeting the open regions in RNA chemistry to enhance the binding of ASO to these target regions and put the closed region in phosphonothioate DNA chemistry. We showed that mixmer increased the specificity by up to 3-fold when compared with the classical gapmer design (Figure 5H). This design approach provides evidence of the value of introducing computational prediction of the secondary structure of the target sequence into the design of gapmer ASOs to enhance its targeting specificity. It is noted that the secondary structure of the target RNA sequence used in this study was only software predicted. Experimental methods for probing RNA secondary structures could be integrated to validate or improve the structural prediction.[45]

The allele-specific silencing effect of mixmer was further improved by introducing a single nucleotide mismatch into the ASO sequence. This resulted in a further 3-fold increase when compared with the original mixmer sequence and a 10-fold improvement compared with the classical gapmer design (Figure 5H). In addition, the efficiency of the best performing MM-ASO (MM (1–7)) showed a detectable restoration of functional collagen VI secretion and localization in ECM in patient-derived dermal fibroblasts (Figure 6).

The introduction of mismatched nucleotides has previously been used in siRNA design to enhance allele discrimination based on a single nucleotide variation between the mutant and wild-type alleles.[46] Introduction of a one-base mismatch into the guide seed region of siRNA significantly influenced allele discrimination and hence allele-specific silencing when tested in targeting the single-nucleotide *COL6A1* c.850G>A (p.G284R) mutation,[34] and the c.877G>A (p.G293R) mutation,[47] responsible for a dominant form of UCMD. The introduction of mismatched nucleotides has also been previously investigated in gapmer ASOs to define the rule of mismatches on RNase H allele-selective cleavage of RNA within DNA/RNA duplexes.[46,48] The mismatch nucleotide may reduce the binding affinity of gapmer ASOs to the WT transcripts when the mismatch is introduced in the RNA wing sequence, or the cleavage ability when the mismatch is introduced in the DNA gap sequence.[48] In this study, we confirm that introducing a single nucleotide mismatch can further improve the specificity of mixmer ASOs in preferentially targeting the mutant transcripts.

It is widely accepted that the single-stranded gapmer ASOs silence genes by activating the endogenous RNase H through its central PS DNA gap. A similar MOA applies to mixmer ASOs, where the RNA parts function via steric blockade of the target RNA and the DNA parts work via RNase H-mediated cleavage of the DNA/RNA duplex. While double-stranded siRNAs silence target genes by activating the RISC.[49] So far RNase H activation has been considered to be the sole MOA of gapmer ASO gene silencing. In this study, we have confirmed that mixmer ASO, containing at least four consecutive PS DNA nucleotides in each DNA gap, share the same MOA via RNase H activation for target gene silencing, rather than using the steric blockage mechanism (Figures 7C and 7D). However, when using gapmer ASO as a negative control for RISC inhibition assembly studies, we detected the involvement of not only RNase H, but also RISC-induced gene silencing, although to a lesser extent when compared with RNase H activation-mediated gene silencing (Figure 7C). RISC functions in gene silencing for single-stranded RNA fragments such as microRNA or double-stranded siRNA. The single strand of RNA acts as a template for RISC to recognize the mRNA transcript and activates Argonaute (AGO) to cleave the mRNA. It is likely that the duplexes formed after the binding of gapmer ASO to the target mRNA may also be taken up by AGO, which then initiates target mRNA cleavage. Indeed, Ago-2 has previously been reported to be involved in gapmer ASO-induced gene silencing, as a putative cytoplasmic pathway of gapmer ASO. These data suggest that the MOA of gapmers might be more complicated than that has been previously appreciated.[50]

Allele-specific gene silencing approaches by either siRNA or gapmer ASOs are still at early drug developmental stages. This strategy has been extensively investigated in Huntington's disease to improve the non-allele-specific huntingtin (HTT)-lowering gapmer ASO tominersen, of which a phase III trial was stopped due to worsening clinical outcomes.[51] Two other clinical trials were initiated by Wave Life Sciences, SELECT-HD (NCT05032196) and PRECISION-HD2 (NCT03225846), using their proprietary stereopure ASO with wing chemistry in 2′-OMe to target common heterozygous SNPs. However, both trials were halted for failing to lower

concentrations between 10 nM and 100 nM for 24 h with Lipofectamine transfection, and measured at mRNA level by (E) RT-PCR and (F) allele-specific qRT-PCR on WT and Mut COL6A3 transcripts, respectively. Mock, cells treated with Lipofectamine 2000 only; NT, untreated controls. Data were normalized to untreated controls and analyzed by one-way ANOVA and post-Bonferroni test. Data are presented as mean ± SD (*$p \leq 0.05$; **$p \leq 0.01$; ***$p \leq 0.001$). (G) Dose-response curve of M-7 showing IC_{50} based on the quantification of WT and mutCOL6A3 transcripts.

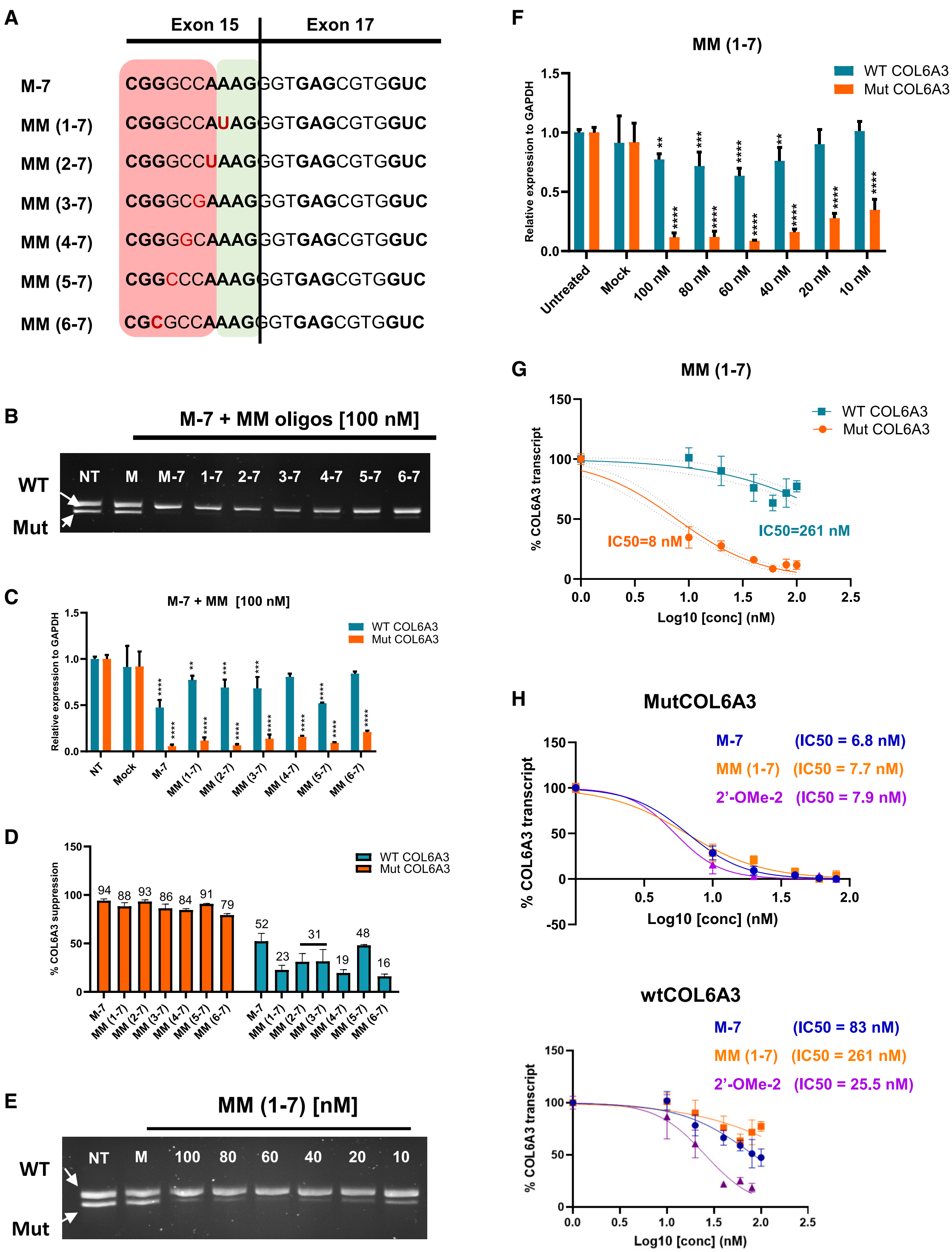

Figure 5. Design and evaluation of MM-ASOs

(A) Schematic illustration of mixmer M-7 and MM-ASOs designed by introducing one mismatch nucleotide (in red). RNA nucleotides are shown in bold and highlighted in gray, red squares highlight nucleotides in closed structure and green squares for nucleotide in opened structures in both wild-type (WT) and Mut sequences. UCMD fibroblasts were treated with M-7 and MM-ASOs at 100 nM for 24 h with Lipofectamine transfection and measured by (B) RT-PCR and (C) allele-specific RT-qPCR on WT and mutCOL6A3. Mock, cells treated with Lipofectamine 2000 only; NT, untreated controls. Data were normalized to untreated controls and analyzed by one-way ANOVA and

(legend continued on next page)

mutant HTT.[52] There is an urgent requirement of a further improved therapeutic strategy to lower toxic HTT. Several allele-specific gene silencing approaches, including ASOs, siRNA, and CRISPR-Cas9 gene editing, have been explored for treating dominant COL6-related muscular dystrophy.[30,31,33,34,53] Although these approaches show promise, there is still a strong need for further investigations to enhance the efficiency and specificity. The minimum percentage of mutant allele silencing to elicit clinical benefit is expected to vary depending on the specific genes or mutations involved. Recent research in COL6-CMD indicates that, while the minimum level of mutant transcripts silencing remains to be determined, complete silencing may not be necessary to restore collagen VI secretion and deposition.[47] Evidence suggests that even partial reduction of mutant transcripts could confer clinical benefit, as evidenced by patients with somatic mosaicism of severe UCMD variants who present only mild Bethlem phenotype.[54,55] Nevertheless, our studies provide a novel design principle for allele-specific ASOs, aiming to enhance silencing specificity, based on the target mRNA's secondary structures and the introduction of nucleotide mismatching. By addressing these design aspects, our findings contribute valuable insights to the ongoing efforts in optimizing allele-specific gene silencing strategies for enhanced therapeutic outcomes in COL6-related muscular dystrophy.

MATERIALS AND METHODS

ASOs

All ASOs were synthetized by Eurogentec Ltd. RNA Folding Form V2.3 (http://www.unafold.org/mfold/applications/rna-folding-form-v2.php) has been used for secondary structure predictions.[56] For both classical gapmer and mixmer ASOs, OligoAnalyzerTM Tool was used to analyze GC % and Tm (https://eu.idtdna.com/pages/tools/oligoanalyzer). GGGenome was used for off-target sequences detection (https://gggenome.dbcls.jp/) and RNAup web server was used to calculate the ASOs binding affinity with the target sequences (http://rna.tbi.univie.ac.at/cgi-bin/RNAWebSuite/RNAup.cgi).

Patients and *COL6A3* pathogenic variant

This study was conducted in accordance with the Declaration of Helsinki. Written informed consent was obtained from all participants, including patients and control subjects, for the utilization of skin biopsy samples. Primary fibroblasts were established from skin biopsies from two patients carrying c.6210+1G>A variant in *COL6A3* gene (NM_004369.4), as well as one control subject (pediatric subject with no pathogenic variant in *COL6A* genes).

The pathogenic variant under investigation in this study is a common dominant mutation in *COL6A3* gene (c.6210+1G>A), which leads to the in-frame skipping of exon 16 in the mature transcripts of *COL6A3*. The exon 16 skipping was confirmed in both patients through cDNA product sequencing.

Cell culture

Established skin fibroblast lines were supplied by the MRC Center for Neuromuscular Disease Biobank London (REC reference number 06/Q0406/33, ethics number 13/LO/1894). Fibroblasts were cultured in DMEM (Thermo Fisher Scientific) supplemented with 10% fetal bovine serum (FBS; Thermo Fisher Scientific) at 37°C in 5% CO_2.

In vitro transfection of ASOs and siRNAs for RNA analysis

The *in vitro* testing of gapmer and mixmer ASOs, and siRNA were conducted using Lipofectamine 2000 (Invitrogen) transfection reagent, following the manufacturer's instructions. Cells were seeded in six-well plates at a density of 2×10^5 per well in growth medium, aiming to reach 80% confluence on the following day. The day after, ASOs (Table 1) and siRNA-A3 (Cat# hs.Ri.COL6A3.13.1, IDT) were mixed with 5 μL/mL Lipofectamine 2000 in Opti-MEM medium (Thermo Fisher Scientific), and cells were transfected and incubated at 37°C in 5% CO_2. After 24 h, the cells were harvested for RNA analysis.

In vitro transfection of ASOs for protein analysis

Collagen-precoated glass coverslips (Corning) were placed in individual wells of six-well plates, and cells were seeded at a density of 1×10^5 per well in growth medium. The following day, ASOs were mixed with Lipofectamine 2000 at a concentration of 5 μL/mL in Opti-MEM medium and the cells were transfected and incubated at 37°C in 5% CO_2. After 24 h, the medium was replaced with fresh growth medium supplemented with 50 mg/mL of L-ascorbic acid (Cat# 013–12061, Alpha Laboratories), and cells were further incubated for 24 h,[27] followed by immunofluorescence analysis.

RNA isolation, complementary DNA synthesis, PCR, and Sanger sequencing

Total RNA from fibroblasts was isolated using RNeasy mini kit (Qiagen) according to the manufacturer's instructions. The quality and quantity of RNA samples were assessed using a NanoDrop spectrophotometer (Thermo Fisher Scientific). RNA was reverse transcribed using Applied Biosystems High-Capacity RNA-to-cDNA Kit (Thermo Fisher Scientific) according to manufacturer's recommendation. To evaluate the efficiency of ASOs, PCR was conducted using a primer set designed to amplify both wild-type (275 bp) and mutant (221 bp) transcripts. The forward and reverse primers were 5′-GAG CAG CTT GAC AAC ATT GC-3′ and 5′-CCA ATT TCT CCT ACT TCG CCC-3′, respectively. The

post-Bonferroni test. Data are presented as mean ± SD (*$p \le 0.05$; **$p \le 0.01$; ***$p \le 0.001$). (D) Average of the mutant (in orange) and wild-type (in blue) transcripts suppression after treatment with M-7 and MMs. (E) UCMD fibroblasts were treated with MM (1–7) ASO at a range of concentrations between 10 nM and 100 nM for 24 h with Lipofectamine transfection, and measured at mRNA level by (E) RT-PCR and (F) allele-specific RT-qPCR on WT and Mut COL6A3 transcripts. Mock, cells treated with Lipofectamine 2000 only; NT, untreated controls. Data were normalized to untreated controls and analyzed by one-way ANOVA and post-Bonferroni test. Data are presented as mean ± SD (*$p \le 0.05$; **$p \le 0.01$; ***$p \le 0.001$. (G) Dose-response curve of MM (1–7) showing IC_{50} based on the quantification of WT and mutCOL6A3 transcripts. (H) Dose-response curve of M-7, MM (1–7), and 2′-OMe-2 showing IC_{50} based on the quantification of WT and mutCOL6A3 transcripts.

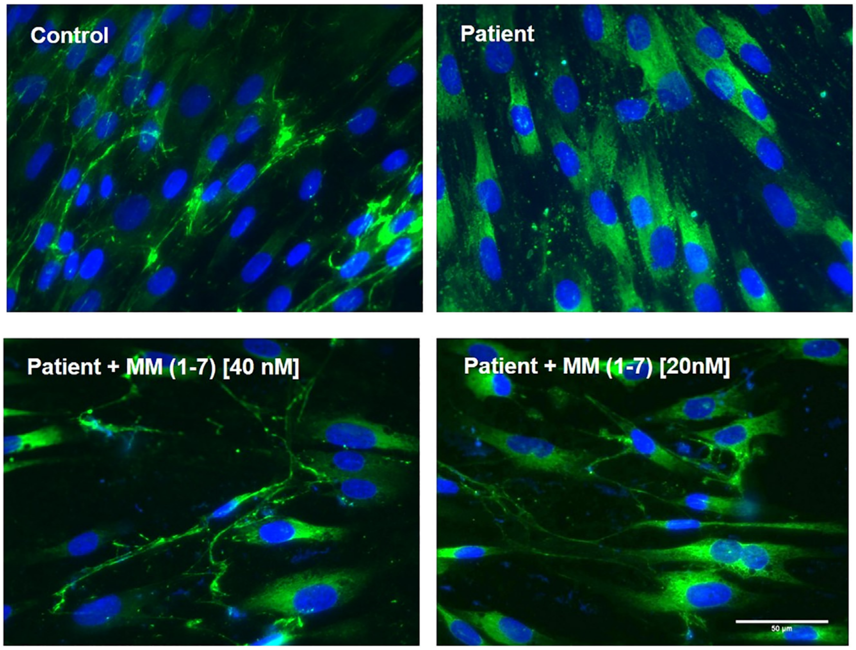

Figure 6. Evaluation of MM (1–7) on restoring collagen VI protein in ECM

Representative images of immunofluorescence staining of collagen VI protein (in green) and nuclei (in blue) in healthy control and patient skin fibroblasts treated with MM (1–7). Pictures were captured under fluorescence microscopy at 40× magnification. Scale bars, 100 μm.

PCR protocol consisted of an initial denaturation at 94°C for 3 min, followed by denaturation at 94°C for 45 s, annealing at 55°C for 30 s, and extension at 72°C for 90 s. These steps were repeated for a total of 30 cycles, with a final extension at 72°C for 10 min. The PCR products were visualized by electrophoresis on a 1.5% agarose gel and imaged using the Gel Doc XR imaging system (Bio-Rad). Additionally, 5 μL of each amplicon were purified for Sanger sequencing using both forward and reverse primers.

Quantitative real-time PCR

Quantitative real-time PCR was performed using the Takyon Rox SYBR qPCR kit (Eurogentec) with 10 ng cDNA as a template. Several primer sets were used in the analysis: one set designed to specifically amplify the wild-type *COL6A3* transcripts (wtCOL6A3) (forward 5′- ATC GGG CCA AAG GGT ATT -3′ and reverse 5′-CCG AGA GCC CTT TAC TCC TC-3′), a second set to specifically amplify the mut*COL6A3* transcripts (mutCOL6A3) (forward 5′-ATC GGG CCA AAG GGT GA-3′ and reverse 5′-ATC CAG ACC ATC CAG TCC AA-3′), and the third set to amplify glyceraldehyde 3-phosphate dehydrogenase (*GAPDH*) (forward 5′-TTG AGG TCA ATG AAG GGG TC-3′ and reverse 5′-GAA GGT GAA GGT CGG AGT CA-3′). The StepOne real-time PCR system (Applied Biosystems) was utilized for the analysis using the following program: activation at 95°C for 2 min, 40 cycles of denaturation at 95°C for 10 s, and annealing/extension at 60°C for 30 s. The fold changes of mutCOL6A3 and wtCOL6A3 were determined by the $2^{-\Delta\Delta Ct}$ method, relative to untreated samples and normalized to the reference *GAPDH* gene.

ATA assay

To investigate the mechanism responsible for the silencing of the target mRNA induced by mixmer ASOs (RNase H1 or RISC), an inhibitor of the RISC was employed. ATA (Sigma) was used as a molecule capable of inhibiting the binding between RNA and Ago2, as well as the *de novo* RISC assembly.[44] Fibroblasts were treated with siRNA-A3, a classical gapmer ASO (LNA-2) and a mixmer ASO (MM (1–7)). Transfection of fibroblasts was performed for 24 h using Lipofectamine 2000 as the transfection reagent, with 5 nM of siRNA-A3, or 100 nM of gapmer or mixmer ASO. After 24 h, 6.25 μM ATA inhibitor or DMSO were added in control and treated samples. The cells were then incubated at 37°C in 5% CO_2. After an additional 24 h, fibroblasts were harvested for RNA analysis.

RNase H assay

Complementary COL6A3 mutant RNA sequences (5′-rUrCrGrGrGrCrCrArArArGrGrGrUrGrArGrCrGrUrGrGrUrC-3′) of 2′-OMe-2, LNA-2, M-1. and MM (1–7) were synthesized by IDT and used for duplex formation. RNA COL6A3 sequence was annealed with gapmer or mixmers oligos to form the RNA/ASO duplexes.

We mixed 5 μL 100 μM RNA and 5 μL 100 μM of each oligos with 12.5 μL 10× PBS and 32.5 μL nuclease-free H_2O. Samples were heated to 95°C for 10 min before cooling down at approximately 1 °C/min for 50 min. The cleavage assay was performed according to the manufacturer's instructions using RNase H enzyme (NEB #M0297) and the reaction was stopped by adding 1 μL of 0.5 M EDTA. Samples were run on 20% TBE gel (Thermo Fisher Scientific) using microRNA marker (NEB) as the RNA size marker. The intensity of each band was quantified using ImageJ software.

Immunofluorescence staining

Immunofluorescence staining was performed in control and patient cell lines following incubation with L-ascorbic acid at 50 mg/mL. After removing the medium, cells on coverslips were washed once with PBS and fixed for 10 min at room temperature (RT) with 4% paraformaldehyde (Thermo Fisher Scientific). Subsequentially, cells were rinsed with PBS and incubated for 1 h at RT in a blocking solution containing 5% goat serum in PBS with 0.05% Triton. Next, cells were incubated with mouse anti-human COL6 antibody (Cat# MAB1944, Merck) at a dilution of 1:2,000 for 1 h at RT. Following antibody incubation, cells were washed three times for 5 min each

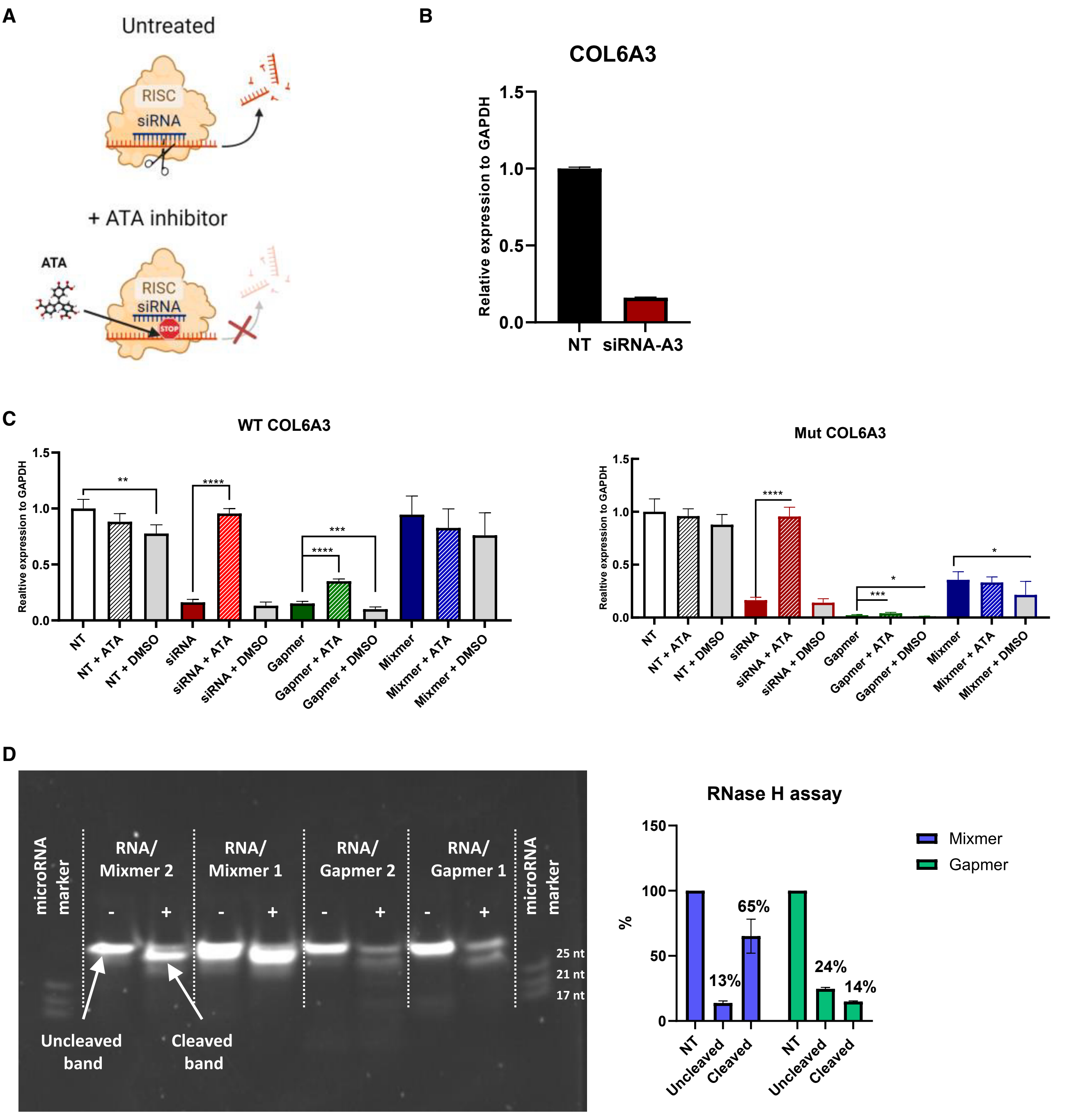

Figure 7. Studies on the underlying mechanism of action of mixmer in gene silencing

(A) Schematic illustration on the mechanism of ATA inhibitor in blocking the RISC formation in siRNA-mediated gene silencing. Image created with BioRender.com. (B) RT-qPCR in samples treated with siRNA-A3 at 10 nM as a positive control. (C) UCMD fibroblasts treated with siRNA-A3 (in red), gapmer LNA-2 (in green), MM (1–7) (in blue) were co-treated with either ATA inhibitor at 6.25 μM or DMSO. MutCOL6A3 and wtCOL6A3 transcripts were measured by RT-qPCR. Data were analyzed by one-way ANOVA and post-Bonferroni test and presented as mean ± SD (*p = 0.05; **p = 0.01; ***p = 0.001). (D) The RNase H cleavage assay was conducted on RNA/Gapmer and RNA/Mixmer duplexes. (+) indicates samples that have been incubated with RNase H enzyme, while (–) indicates samples without RNase H enzyme. Gapmer 1 refers to 2′-OMe-2, Gapmer 2 refers to LNA-2, Mixmer 1 refers to M-1 and Mixmer 2 refers to MM(1–7). Please refer to Table 1 for more detail in ASO sequences and different chemical modifications.

with PBS with 0.05% Triton and incubated with secondary antibody (goat-anti mouse Alexa Fluor 488 Cat# A11029, Thermo Fisher Scientific) at a dilution of 1:500. After secondary antibody incubation, cells were washed three times for 5 min each with PBS with 0.05% Triton followed by Hoechst 33342 (Invitrogen) staining. Finally, the coverslips were mounted with hydromount (National Diagnostics), and cells were visualized using a Leica DMR fluorescence microscope.

Statistics

GraphPad Prism version 6.0 was used for statistical analysis and graphs design. Data were analyzed using one-way ANOVA and post-Bonferroni test and presented as mean ± SD. Differences were considered to be statistically significant at $^{*}p \leq 0.05$, $^{**}p \leq 0.01$, and $^{***}p \leq 0.001$.

DATA AND CODE AVAILABILITY

Please contact the corresponding author for data requests.

SUPPLEMENTAL INFORMATION

Supplemental information can be found online at https://doi.org/10.1016/j.omtn.2024.102237.

ACKNOWLEDGMENTS

This work was supported by Muscular Dystrophy UK (19GRO-PG36-0293 and 22GRO-PG36-0552), UKRI MRC (MR/X008029/1 and MR/Y008405/1) and the National Institutes of Health Research Biomedical Research Centre at Great Ormond Street Hospital for Children NHS Foundation Trust and University College London. The MRC Centre for Neuromuscular Diseases Biobank, supported by the National Institute for Health Research Biomedical Research Centers at Great Ormond Street Hospital for Children NHS Foundation Trust at University College London Hospitals NHS Foundation Trust and University College London, is acknowledged for providing patients' fibroblast cell lines. The Muscular Dystrophy UK support to the Dubowitz Neuromuscular Centre is also gratefully acknowledged. The authors would like to acknowledge the networking grant supported by the European Cooperation in Science and Technology (COST) Action CA17103 on Delivery of Antisense RNA Therapeutics (DARTER). The authors would like to acknowledge Dr. Carsten Bonnemann, Dr. Veronique Bolduc, and Mr. Fady Guirguis for their scientific advice. We would also like to thank Prof. Jennifer Morgan and Dr. Silvia Torelli for critical reading and comments.

AUTHOR CONTRIBUTIONS

S.A., H.Z., and F.M., conceptualized and designed the study and experiments; S.A. and H.Z., designed the antisense oligomers; S.A. and S.C. performed and analyzed the experiments; P.A. established fibroblast lines from patients' skin biopsies; S.A., S.B., and H.Z., designed the graphical abstract; S.A., H.Z., and F.M. wrote the manuscript.

DECLARATION OF INTERESTS

The authors declare no conflict of interest for this work.

REFERENCES

1. Roberts, T.C., Langer, R., and Wood, M.J.A. (2020). Advances in oligonucleotide drug delivery. Nat. Rev. Drug Discov. *19*, 673–694.
2. Amanat, M., Nemeth, C.L., Fine, A.S., Leung, D.G., and Fatemi, A. (2022). Antisense oligonucleotide therapy for the nervous system: from bench to bedside with emphasis on pediatric neurology. Pharmaceutics *14*, 2389.
3. de Smet, M.D., Meenken, C.J., and van den Horn, G.J. (1999). Fomivirsen – a phosphorothioate oligonucleotide for the treatment of CMV retinitis. Ocul. Immunol. Inflamm. *7*, 189–198.
4. Geary, R.S., Henry, S.P., and Grillone, L.R. (2002). Fomivirsen: clinical pharmacology and potential drug interactions. Clin. Pharmacokinet. *41*, 255–260.
5. Hair, P., Cameron, F., and McKeage, K. (2013). Mipomersen sodium: first global approval. Drugs *73*, 487–493.
6. Kastelein, J.J.P., Wedel, M.K., Baker, B.F., Su, J., Bradley, J.D., Yu, R.Z., Chuang, E., Graham, M.J., and Crooke, R.M. (2006). Potent reduction of apolipoprotein B and low-density lipoprotein cholesterol by short-term administration of an antisense inhibitor of apolipoprotein B. Circulation *114*, 1729–1735.
7. Gales, L. (2019). Tegsedi (Inotersen): an antisense oligonucleotide approved for the treatment of adult patients with hereditary transthyretin amyloidosis. Pharmaceuticals *12*, 78.
8. Paik, J., and Duggan, S. (2019). Volanesorsen: first global approval. Drugs *79*, 1349–1354.
9. Esan, O., and Wierzbicki, A.S. (2020). Volanesorsen in the treatment of familial chylomicronemia syndrome or hypertriglyceridaemia: design, development and place in therapy. Drug Des. Dev. Ther. *14*, 2623–2636.
10. Blair, H.A. (2023). Tofersen: first approval. Drugs *83*, 1039–1043.
11. Helm, J., Schöls, L., and Hauser, S. (2022). Towards personalized allele-specific antisense oligonucleotide therapies for toxic gain-of-function neurodegenerative diseases. Pharmaceutics *14*, 1708.
12. (2022). Wave Life Sciences announces positive update from phase 1b/2a SELECT-HD trial with initial results indicating allele-selective target engagement with WVE-003 in Huntington's disease. www.wavelifesciences.com.
13. Liu, Y., Andreucci, A., Iwamoto, N., Yin, Y., Yang, H., Liu, F., Bulychev, A., Hu, X.S., Lin, X., Lamore, S., et al. (2022). Preclinical evaluation of WVE-004, aninvestigational stereopure oligonucleotide forthe treatment of C9orf72-associated ALS or FTD. Mol. Ther. Nucleic Acids *28*, 558–570.
14. Carroll, J.B., Warby, S.C., Southwell, A.L., Doty, C.N., Greenlee, S., Skotte, N., Hung, G., Bennett, C.F., Freier, S.M., and Hayden, M.R. (2011). Potent and selective antisense oligonucleotides targeting single-nucleotide polymorphisms in the Huntington disease gene/allele-specific silencing of mutant huntingtin. Mol. Ther. *19*, 2178–2185.
15. Gagnon, K.T., Pendergraff, H.M., Deleavey, G.F., Swayze, E.E., Potier, P., Randolph, J., Roesch, E.B., Chattopadhyaya, J., Damha, M.J., Bennett, C.F., et al. (2010). Allele-selective inhibition of mutant huntingtin expression with antisense oligonucleotides targeting the expanded CAG repeat. Biochemistry *49*, 10166–10178.
16. Hu, J., Matsui, M., Gagnon, K.T., Schwartz, J.C., Gabillet, S., Arar, K., Wu, J., Bezprozvanny, I., and Corey, D.R. (2009). Allele-specific silencing of mutant huntingtin and ataxin-3 genes by targeting expanded CAG repeats in mRNAs. Nat. Biotechnol. *27*, 478–484.
17. Østergaard, M.E., Southwell, A.L., Kordasiewicz, H., Watt, A.T., Skotte, N.H., Doty, C.N., Vaid, K., Villanueva, E.B., Swayze, E.E., Bennett, C.F., et al. (2013). Rational design of antisense oligonucleotides targeting single nucleotide polymorphisms for potent and allele selective suppression of mutant Huntingtin in the CNS. Nucleic Acids Res. *41*, 9634–9650.
18. Conroy, F., Miller, R., Alterman, J.F., Hassler, M.R., Echeverria, D., Godinho, B.M.D.C., Knox, E.G., Sapp, E., Sousa, J., Yamada, K., et al. (2022). Chemical engineering of therapeutic siRNAs for allele-specific gene silencing in Huntington's disease models. Preprint at bioRxiv. https://doi.org/10.1101/2022.06.29.498088.
19. Giorgio, E., Lorenzati, M., Rivetti di Val Cervo, P., Brussino, A., Cernigoj, M., Della Sala, E., Bartoletti Stella, A., Ferrero, M., Caiazzo, M., Capellari, S., et al. (2019). Allele-specific silencing as treatment for gene duplication disorders: Proof-of-principle in autosomal dominant leukodystrophy. Brain *142*, 1905–1920.

20. Southwell, A.L., Skotte, N.H., Kordasiewicz, H.B., Østergaard, M.E., Watt, A.T., Carroll, J.B., Doty, C.N., Villanueva, E.B., Petoukhov, E., Vaid, K., et al. (2014). In vivo evaluation of candidate allele-specific mutant huntingtin gene silencing antisense oligonucleotides. Mol. Ther. *22*, 2093–2106.

21. Miller, V.M., Xia, H., Marrs, G.L., Gouvion, C.M., Lee, G., Davidson, B.L., and Paulson, H.L. (2003). Allele-specific silencing of dominant disease genes. Proc. Natl. Acad. Sci. USA *100*, 7195–7200.

22. (2021). Wave Life Sciences Provides update on phase 1b/2a PRECISION-HD trials additional results from the PRECISION-HD Trials mHTT assessments. www.wavelifesciences.com.

23. Lamandé, S.R., and Bateman, J.F. (2018). Collagen VI disorders: Insights on form and function in the extracellular matrix and beyond. Matrix Biol. *71–72*, 348–367.

24. Cescon, M., Gattazzo, F., Chen, P., and Bonaldo, P. (2015). Collagen VI at a glance. J. Cell Sci. *128*, 3525–3531.

25. Allamand, V., Briñas, L., Richard, P., Stojkovic, T., Quijano-Roy, S., and Bonne, G. (2011). ColVI myopathies: Where do we stand, where do we go? Skelet. Muscle *1*, 30.

26. Tagliavini, F., Pellegrini, C., Sardone, F., Squarzoni, S., Paulsson, M., Wagener, R., Gualandi, F., Trabanelli, C., Ferlini, A., Merlini, L., et al. (2014). Defective collagen VI α6 chain expression in the skeletal muscle of patients with collagen VI-related myopathies. Biochim. Biophys. Acta *1842*, 1604–1612.

27. Jimenez-Mallebrera, C., Maioli, M.A., Kim, J., Brown, S.C., Feng, L., Lampe, A.K., Bushby, K., Hicks, D., Flanigan, K.M., Bonnemann, C., et al. (2006). A comparative analysis of collagen VI production in muscle, skin and fibroblasts from 14 Ullrich congenital muscular dystrophy patients with dominant and recessive COL6A mutations. Neuromuscul. Disord. *16*, 571–582.

28. Aguti, S., Bolduc, V., Ala, P., Turmaine, M., Bönnemann, C.G., Muntoni, F., and Zhou, H. (2020). Exon-Skipping Oligonucleotides Restore Functional Collagen VI by Correcting a Common COL6A1 Mutation in Ullrich CMD. Mol. Ther. Nucleic Acids *21*, 205–216.

29. Bolduc, V., Foley, A.R., Solomon-Degefa, H., Sarathy, A., Donkervoort, S., Hu, Y., Chen, G.S., Sizov, K., Nalls, M., Zhou, H., et al. (2019). A recurrent COL6A1 pseudoexon insertion causes muscular dystrophy and is effectively targeted by splice-correction therapies. JCI Insight *4*, e124403.

30. Gualandi, F., Manzati, E., Sabatelli, P., Passarelli, C., Bovolenta, M., Pellegrini, C., Perrone, D., Squarzoni, S., Pegoraro, E., Bonaldo, P., et al. (2012). Antisense-induced messenger depletion corrects a COL6A2 dominant mutation in Ullrich myopathy. Hum. Gene Ther. *23*, 1313–1318.

31. Marrosu, E., Ala, P., Muntoni, F., and Zhou, H. (2017). Gapmer antisense oligonucleotides suppress the mutant allele of COL6A3 and restore functional protein in Ullrich muscular dystrophy. Mol. Ther. Nucleic Acids *8*, 416–427.

32. Aguti, S., Marrosu, E., Muntoni, F., and Zhou, H. (2020). Gapmer antisense oligonucleotides to selectively suppress the mutant allele in COL6A genes in dominant Ullrich congenital muscular dystrophy. Methods Mol. Biol. *2176*, 221–230.

33. Bolduc, V., Zou, Y., Ko, D., and Bönnemann, C.G. (2014). SiRNA-mediated allele-specific silencing of a COL6A3 mutation in a cellular model of dominant Ullrich muscular dystrophy. Mol. Ther. Nucleic Acids *3*, e147.

34. Naguchi, S., Ogawa, M., Kawahara, G., Malicdan, M.C., and Nishino, I. (2014). Allele-specific gene silencing of mutant mRNA restores cellular function in Ullrich congenital muscular dystrophy fibroblast. Mol. Ther. Nucleic Acids *3*, e171.

35. Monia, B.P., Lesnik, E.A., Gonzalez, C., Lima, W.F., McGee, D., Guinosso, C.J., Kawasaki, A.M., Cook, P.D., and Freier, S.M. (1993). Evaluation of 2'-modified Oligonucleotides Containing 2'-deoxy Gaps as Antisense Inhibitors of Gene Expression, *266*, pp. 14514–14522.

36. Wu, H., Lima, W.F., Zhang, H., Fan, A., Sun, H., and Crooke, S.T. (2004). Determination of the role of the human RNase H1 in the pharmacology of DNA-like antisense drugs. J. Biol. Chem. *279*, 17181–17189.

37. Dhuri, K., Bechtold, C., Quijano, E., Pham, H., Gupta, A., Vikram, A., and Bahal, R. (2020). Antisense oligonucleotides: An emerging area in drug discovery and development. J. Clin. Med. *9*, 2004–2024.

38. Touznik, A., Maruyama, R., Hosoki, K., Echigoya, Y., and Yokota, T. (2017). LNA/DNA mixmer-based antisense oligonucleotides correct alternative splicing of the SMN2 gene and restore SMN protein expression in type 1 SMA fibroblasts. Sci. Rep. *7*, 3672.

39. Le, B.T., Raguraman, P., Kosbar, T.R., Fletcher, S., Wilton, S.D., and Veedu, R.N. (2019). Antisense oligonucleotides targeting angiogenic factors as potential cancer therapeutics. Mol. Ther. Nucleic Acids *14*, 142–157.

40. Hillebrand, F., Ostermann, P.N., Müller, L., Degrandi, D., Erkelenz, S., Widera, M., Pfeffer, K., and Schaal, H. (2019). Gymnotic delivery of LNA mixmers targeting viral SREs induces HIV-1 mRNA degradation. Int. J. Mol. Sci. *20*, 1088.

41. Hagedorn, P.H., Persson, R., Funder, E.D., Albæk, N., Diemer, S.L., Hansen, D.J., Møller, M.R., Papargyri, N., Christiansen, H., Hansen, B.R., et al. (2018). Locked nucleic acid: modality, diversity, and drug discovery. Drug Discov. Today *23*, 101–114.

42. Aartsma-Rus, A., van Vliet, L., Hirschi, M., Janson, A.A.M., Heemskerk, H., de Winter, C.L., de Kimpe, S., van Deutekom, J.C.T., 't Hoen, P.A., and van Ommen, G.J.B. (2009). Guidelines for antisense oligonucleotide design and insight into splice-modulating mechanisms. Mol. Ther. *17*, 548–553.

43. Vickers, T.A., Wyatt, J.R., and Freier, S.M. (2000). Effects of RNA secondary structure on cellular antisense activity. Nucleic Acids Res. *28*, 1340–1347.

44. Tan, G.S., Chiu, C.H., Garchow, B.G., Metzler, D., Diamond, S.L., and Kiriakidou, M. (2012). Small molecule inhibition of RISC loading. ACS Chem. Biol. *7*, 403–410.

45. Hu, X., Wu, Y., Lu, Z.J., and Yip, K.Y. (2016). Analysis of sequencing data for probing RNA secondary structures and protein-RNA binding in studying posttranscriptional regulations. Briefings Bioinf. *17*, 1032–1043.

46. Ohnishi, Y., Tamura, Y., Yoshida, M., Tokunaga, K., and Hohjoh, H. (2008). Enhancement of allele discrimination by introduction of nucleotide mismatches into siRNA in allele-specific gene silencing by RNAi. PLoS One *3*, e2248.

47. Brull, A., Sarathy, A., Bolduc, V., Chen, G.S., McCarty, R.M., and Bönnemann, C.G. (2024). Optimized allele-specific silencing of the dominant-negative COL6A1 G293R substitution causing collagen VI-related dystrophy. Mol. Ther. Nucleic Acids *35*, 102178.

48. Magner, D., Biala, E., Lisowiec-Wachnicka, J., and Kierzek, R. (2017). Influence of mismatched and bulged nucleotides on SNP-preferential RNase H cleavage of RNA-antisense gapmer heteroduplexes. Sci. Rep. *7*, 12532.

49. Watts, J.K., and Corey, D.R. (2014). Gene silencing by siRNAs and antisense oligonucleotides in the laboratory and the clinic. J. Pathol. *226*, 365–379.

50. Castanotto, D., Lin, M., Kowolik, C., Wang, L., Ren, X.Q., Soifer, H.S., Koch, T., Hansen, B.R., Oerum, H., Armstrong, B., et al. (2015). A cytoplasmic pathway for gapmer antisense oligonucleotide mediated gene silencing in mammalian cells. Nucleic Acids Res. *43*, 9350–9361.

51. Tabrizi, S.J., Leavitt, B.R., Landwehrmeyer, G.B., Wild, E.J., Saft, C., Barker, R.A., Blair, N.F., Craufurd, D., Priller, J., Rickards, H., et al. (2019). Targeting huntingtin expression in patients with Huntington's disease. N. Engl. J. Med. *380*, 2307–2316.

52. Rook, M.E., and Southwell, A.L. (2022). Antisense oligonucleotide therapy: from design to the Huntington disease clinic. BioDrugs *36*, 105–119.

53. López-Márquez, A., Morín, M., Fernández-Peñalver, S., Badosa, C., Hernández-Delgado, A., Natera-de Benito, D., Ortez, C., Nascimento, A., Grinberg, D., Balcells, S., et al. (2022). CRISPR/Cas9-mediated allele-specific disruption of a dominant COL6A1 pathogenic variant improves collagen VI network in patient fibroblasts. Int. J. Mol. Sci. *23*, 4410.

54. D'Amico, A., Fattori, F., Tasca, G., Petrini, S., Gualandi, F., Bruselles, A., D'Oria, V., Verardo, M., Carrozzo, R., Niceta, M., et al. (2017). Somatic mosaicism represents an underestimated event underlying collagen 6-related disorders. Eur. J. Paediatr. Neurol. *21*, 873–883.

55. Donkervoort, S., Hu, Y., Stojkovic, T., Voermans, N.C., Foley, A.R., Leach, M.E., Dastgir, J., Bolduc, V., Cullup, T., de Becdelièvre, A., et al. (2015). Mosaicism for dominant collagen 6 mutations as a cause for intrafamilial phenotypic variability. Hum. Mutat. *36*, 48–56.

56. Zuker, M. (2003). Mfold web server for nucleic acid folding and hybridization prediction. Nucleic Acids Res. *31*, 3406–3415.

OMTN, Volume *35*

Supplemental information

Strategies to improve the design of gapmer antisense oligonucleotide on allele-specific silencing

Sara Aguti, Shuzhi Cheng, Pierpaolo Ala, Sean Briggs, Francesco Muntoni, and Haiyan Zhou

Table S1: R^2 values.

R^2 value for dose-response study using 2’-OMe, 2’-MOE, LNA oligos, mixmer and mismatch-mixmer oligos.

	**2'-OMe-1**	**2'-OMe-2**	**2'-OMe-3**	
**WT COL6A3**	0.8541	0.9035	0.7723	
**Mut COL6A3**	0.9772	0.9439	0.9853	
	**2'-MOE-2**	**2'-MOE-3**		
**WT COL6A3**	0.8478	0.9714		
**Mut COL6A3**	0.9696	0.9968		
	**LNA-1**	**LNA-2**		
**WT COL6A3**	0.7882	0.8078		
**Mut COL6A3**	0.8066	0.9382		
	**M-1**	**M-2**	**M-3**	**M-4**
**WT COL6A3**	0.3765	0.5339	-0.4405	-2.86
**Mut COL6A3**	0.9533	0.9367	0.6478	0.6632
	**M-5**	**M-6**	**M-7**	
**WT COL6A3**	-0.2134	0.07064	0.8294	
**Mut COL6A3**	0.7049	0.7014	0.9719	
	**MM (1-7)**	**MM (2-7)**	**MM (3-7)**	**MM (4-7)**
**WT COL6A3**	0.5299	0.5797	0.4955	0.5687
**Mut COL6A3**	0.9439	0.9843	0.934	0.9807
	**MM (5-7)**	**MM (6-7)**		
**WT COL6A3**	0.5946	0.4145		
**Mut COL6A3**	0.9038	0.9616		

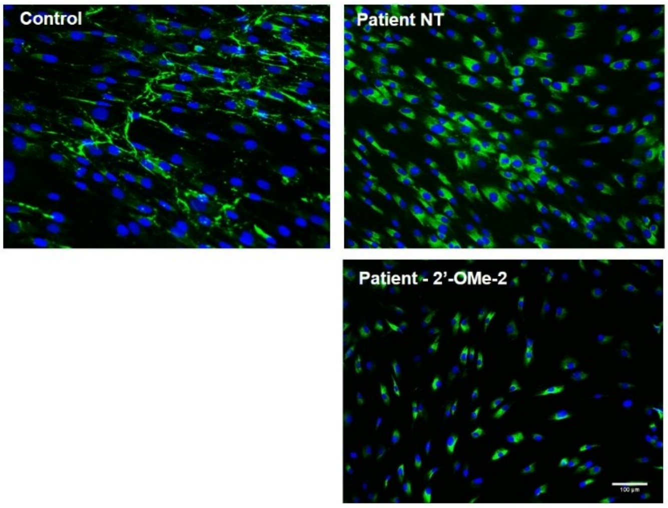

Figure S1: Efficiency of 2'-OMe-2 in restoring ECM collagen VI protein.

Representative images of immunofluorescence staining of collagen VI protein (in green) and nuclei (in blue) in control and patient skin fibroblasts treated with 2'-OMe-2. Pictures were captured under fluorescence microscopy at 10x magnification. Scale bars: 100 µm.

COL6A3 WT

Seq. length		2500 bp	2000bp	1500bp	1000bp	500bp	200bp
**N. structures**		28	31	37	42	12	5
Exon 15	C	0	1	3	7	0	0
	G	0	1	3	7	0	5
	G	24	28	32	33	12	0
	G	0	0	2	6	2	0
	C	0	0	1	2	0	0
	C	3	2	1	0	0	0
	A	0	1	1	1	0	5
	A	24	29	34	40	11	5
	A	24	30	36	42	12	5
	G	24	30	35	37	12	0
Exon 16	G	0	2	4	8	1	0
	G	0	0	0	0	0	0
	U	0	0	1	0	0	0
	A	0	1	2	1	0	0
	U	3	3	5	9	1	5
	U	27	27	30	29	11	0
	C	1	0	0	1	0	0
	C	0	0	0	1	0	0
	U	0	2	1	2	1	0
	G	1	0	0	1	0	0
	G	0	1	5	10	0	0
	A	1	0	3	6	0	0
	G	0	2	4	8	1	0
	A	3	2	3	4	0	3
	A	28	30	35	31	10	5
	G	28	31	35	37	11	0
	A	3	0	1	3	2	0

COL6A3 Δ16

Seq. length		2500 bp	2000bp	1500bp	1000bp	500bp	200bp
**N. structures**		30	30	33	35	19	5
Exon 15	C	1	1	4	5	7	0
	G	12	10	14	18	11	3
	G	0	1	3	5	6	0
	G	0	0	0	0	0	0
	C	7	5	3	1	0	0
	C	0	2	2	7	3	0
	A	12	12	15	25	16	3
	A	20	15	29	35	19	5
	A	20	15	29	34	18	5
	G	8	5	14	17	7	2
Exon 17	G	8	3	13	11	5	2
	G	8	3	13	10	3	2
	U	8	3	13	10	3	2
	G	27	21	32	30	17	5
	A	18	21	23	24	14	2
	G	15	18	20	22	13	2
	C	8	3	13	11	4	2
	G	20	13	27	23	10	5
	U	3	10	3	0	3	0
	G	0	0	2	0	0	0
	G	0	0	1	1	0	0
	U	15	11	10	12	6	3
	C	19	13	12	11	5	3
	C	15	12	14	16	10	3
	G	8	5	9	11	2	0
	C	0	0	0	1	1	0
	C	4	2	2	1	1	0

Figure S2: Heat map on the predicted secondary structures of the targeting mRNA sequences

The number of UNAfold-predicted secondary structures is showed for each sequence length, along with ss-count for wild-type mRNA (left lane) and mutant mRNA (right lane).

Nucleotides were color-coded based on the probability of closed structures: >80% in red, 60-80% in light brown, 40-60% in yellow, 20-40% in light green, and <20% in green.

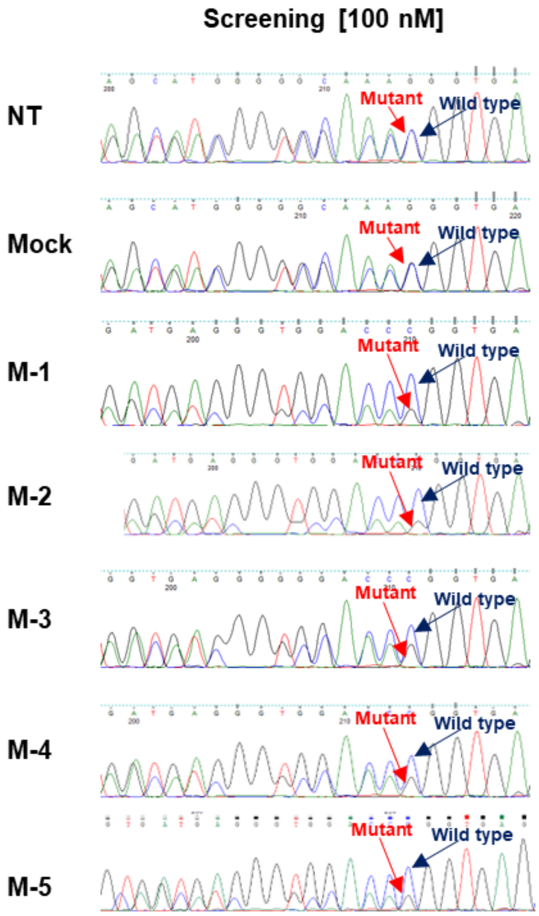

Figure S3: The efficiency of Mixmer ASOs in selectively silencing the mutant allele

Chromatogram of Sanger sequencing of PCR products amplified from UCMD fibroblasts untreated or treated with Mixmer oligos at 100 nM for 24 h. The peaks of wild-type and mutant alleles are indicated with the blue and red arrows.

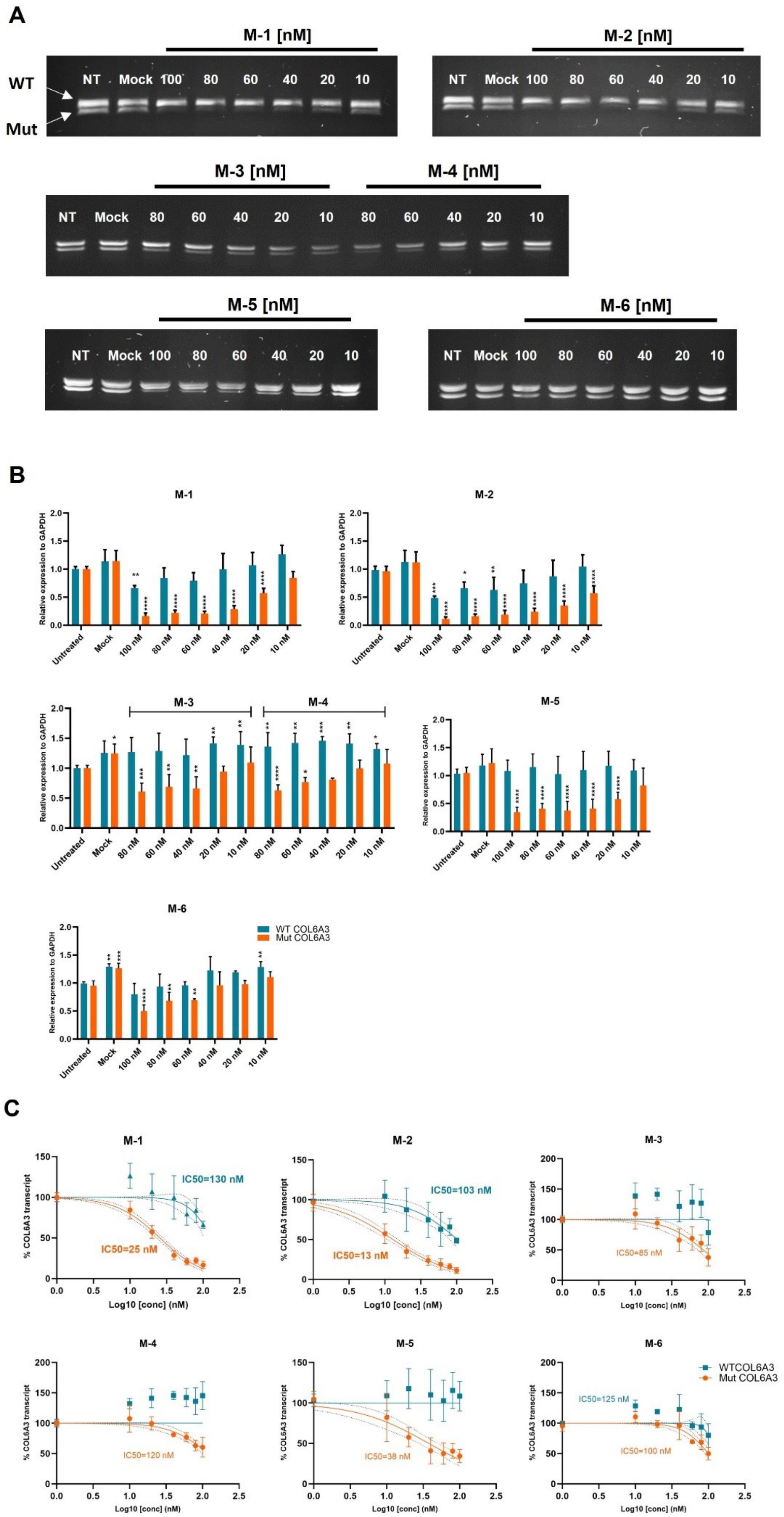
A
M-1 [nM]
M-2 [nM]
WT
Mut
NT
Mock
100
80
60
40
20
10
M-3 [nM]
M-4 [nM]
M-5 [nM]
M-6 [nM]
B
M-1
M-2
M-3
M-4
M-5
M-6
Relative expression to GAPDH
Untreated
Mock
100 nM
80 nM
60 nM
40 nM
20 nM
10 nM
WT COL6A3
Mut COL6A3
C
% COL6A3 transcript
Log10 [conc] (nM)
IC50=130 nM
IC50=25 nM
IC50=103 nM
IC50=13 nM
IC50=85 nM
IC50=120 nM
IC50=38 nM
IC50=125 nM
IC50=100 nM
WTCOL6A3
Mut COL6A3

Figure S4: Dose-response studies of mixmer ASOs on the expression of mutCOL6A3 and wtCOL6A3

UCMD fibroblasts were treated with mixmer ASOs at 100 nM for 24 h, and measured at mRNA level by (A) RT-PCR, (B) allele-specific qRT-PCR on WT and Mut COL6A3 transcripts, respectively. NT= untreated controls, and Mock= cells treated with Lipofectamine 2000 only. Data were normalized to untreated controls and analyzed by one-way ANOVA and post-Bonferroni test. Data are presented as mean ± SD (*$p \leq 0.05$; **$p \leq 0.01$; ***$p \leq 0.001$). (C) Dose-response curve showing IC50 based on the quantification of WT and mutant COL6A3 transcripts.

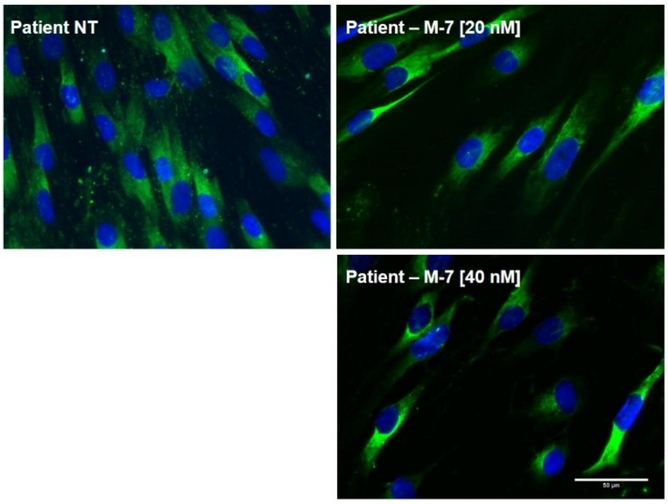

Figure S5: Efficiency of M-7 in restoring ECM collagen VI protein.

Representative images of immunofluorescence staining of COL6 protein (in green) and nuclei (in blue) in untreated patient skin fibroblasts and patient skin fibroblasts treated with M-7 at 20 nM and 40 nM concentration. Fibroblasts were permeabilized using 0.05% Triton X-100. Pictures were captured under fluorescence microscopy at 40x magnification. Scale bars: 50 µm.

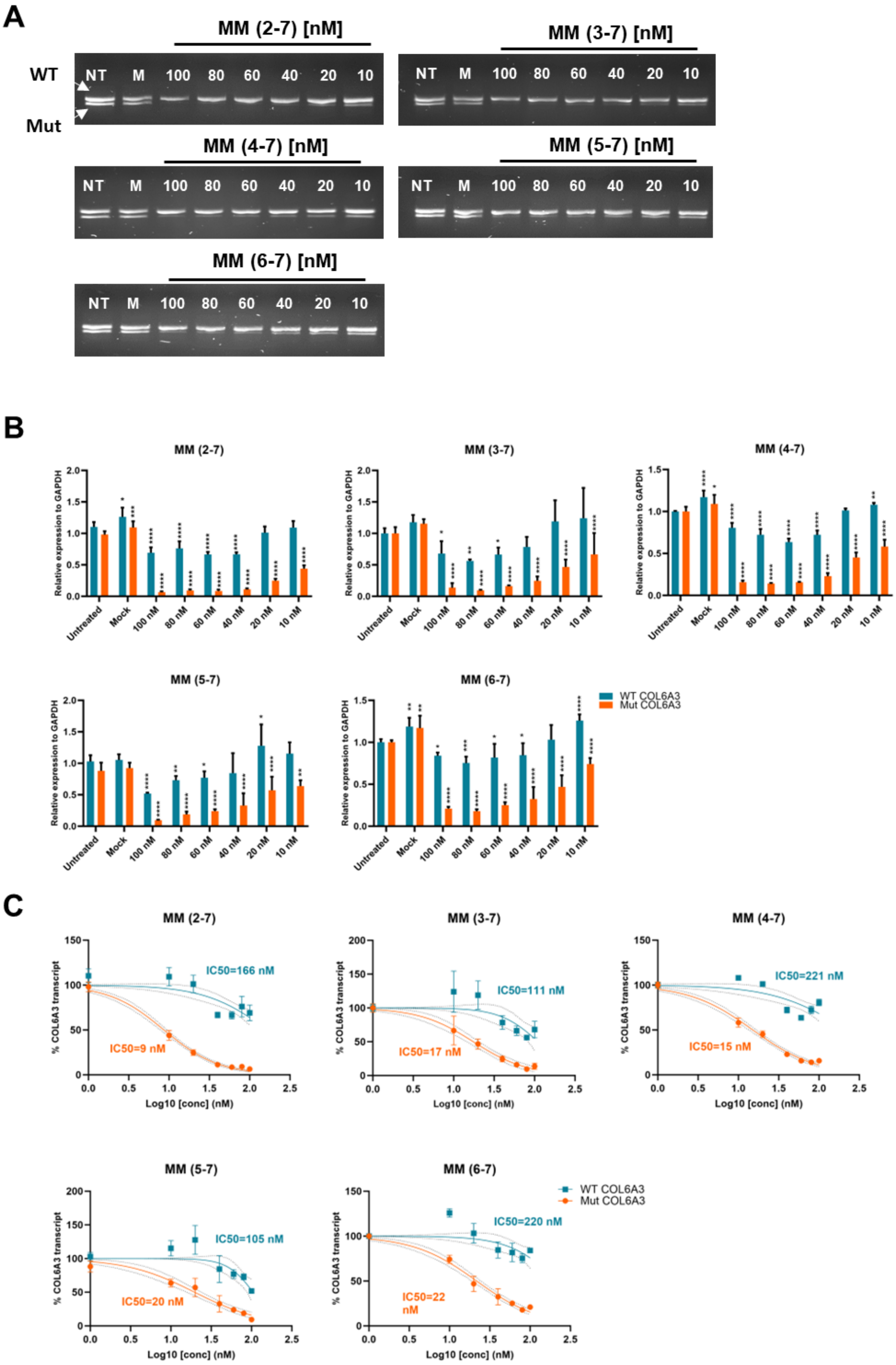

Figure S6: Dose-response studies of mismatch-mixmer ASOs on the expression of mutCOL6A3 and wtCOL6A3.

UCMD fibroblasts were treated with mismatch-mixmer ASOs at 100 nM for 24 h, and measured at mRNA level by (A) RT-PCR, (B) allele-specific qRT-PCR on WT and Mut

COL6A3 transcripts, respectively. NT= untreated controls, and Mock= cells treated with Lipofectamine 2000 only. Data were normalized to untreated controls and analyzed by one-way ANOVA and post-Bonferroni test. Data are presented as mean ± SD ($*p \leq 0.05$; $**p \leq 0.01$; $***p \leq 0.001$). (C) Dose-response curve of showing IC50 based on the quantification of WT and mutant COL6A3 transcripts.
